# Single-cell analysis of long non-coding RNAs in the developing human neocortex

**DOI:** 10.1186/s13059-016-0932-1

**Published:** 2016-04-14

**Authors:** Siyuan John Liu, Tomasz J. Nowakowski, Alex A. Pollen, Jan H. Lui, Max A. Horlbeck, Frank J. Attenello, Daniel He, Jonathan S. Weissman, Arnold R. Kriegstein, Aaron A. Diaz, Daniel A. Lim

**Affiliations:** Department of Neurological Surgery, University of California, San Francisco, Ray and Dagmar Dolby Regeneration Medicine Building, 35 Medical Center Way, RMB 1037, San Francisco, CA 94143 USA; Eli and Edythe Broad Center of Regeneration Medicine and Stem Cell Research, San Francisco, CA 94143 USA; Department of Neurology, San Francisco, CA 94143 USA; Department of Cellular and Molecular Pharmacology, San Francisco, CA 94143 USA; Howard Hughes Medical Institute, San Francisco, CA 94143 USA; California Institute for Quantitative Biomedical Research, San Francisco, CA 94143 USA; Center for RNA Systems Biology, San Francisco, CA 94143 USA; University of California, San Francisco, San Francisco, CA 94143 USA; San Francisco Veterans Affairs Medical Center, San Francisco, CA 94121 USA; Present Address: Department of Biology and Howard Hughes Medical Institute, Stanford University, Stanford, CA 94305 USA

**Keywords:** lncRNA, Single-cell RNA-seq, Developing brain, CRISPRi

## Abstract

**Background:**

Long non-coding RNAs (lncRNAs) comprise a diverse class of transcripts that can regulate molecular and cellular processes in brain development and disease. LncRNAs exhibit cell type- and tissue-specific expression, but little is known about the expression and function of lncRNAs in the developing human brain. Furthermore, it has been unclear whether lncRNAs are highly expressed in subsets of cells within tissues, despite appearing lowly expressed in bulk populations.

**Results:**

We use strand-specific RNA-seq to deeply profile lncRNAs from polyadenylated and total RNA obtained from human neocortex at different stages of development, and we apply this reference to analyze the transcriptomes of single cells. While lncRNAs are generally detected at low levels in bulk tissues, single-cell transcriptomics of hundreds of neocortex cells reveal that many lncRNAs are abundantly expressed in individual cells and are cell type-specific. Notably, *LOC646329* is a lncRNA enriched in single radial glia cells but is detected at low abundance in tissues. CRISPRi knockdown of *LOC646329* indicates that this lncRNA regulates cell proliferation.

**Conclusion:**

The discrete and abundant expression of lncRNAs among individual cells has important implications for both their biological function and utility for distinguishing neural cell types.

**Electronic supplementary material:**

The online version of this article (doi:10.1186/s13059-016-0932-1) contains supplementary material, which is available to authorized users.

## Background

Long non-coding RNAs (lncRNAs), transcripts longer than 200 nt without protein coding potential, comprise upwards of 58,000 genes in the human genome and have important roles in neural development, function, and disease [1–9]. LncRNAs exhibit tissue specific expression, with the brain producing an extraordinary number and diversity of lncRNAs [4, 5, 10, 11]. Furthermore, in the brain, lncRNAs have regionally segregated expression patterns [5, 12] and many lncRNAs are enriched in specific sub-populations of the mouse [13] and human [14] cortex. However, little is known about lncRNA expression and function in the developing human brain.

Current annotations of lncRNAs expressed in the human brain are incomplete, partly due to the use of polyadenylated (polyA) transcript selection and RNA-seq libraries that do not preserve strand information [15, 16]. As a result, non-polyadenylated lncRNAs and antisense lncRNAs – several of which have known biological functions [17–19] – remain poorly described. Furthermore, the expression of lncRNAs in the human brain has not been systematically analyzed at the single-cell level, limiting our understanding of temporal- and cell type-specific lncRNAs.

Bulk tissue studies have suggested that lncRNAs are expressed, on average, at lower levels than mRNAs. It has been unclear whether this is due to uniformly low levels of lncRNAs in all cells, or due to high levels of lncRNAs in subpopulations of cells. In the brain, the latter explanation would suggest that lncRNAs, previously thought to be transcriptional noise, might have highly specialized roles in the differentiation or function of specific cell types. While studies of cultured cells provide evidence for both possibilities [20, 21], whether these observations are consistent with the in vivo expression of lncRNAs in cells within heterogeneous tissues – such as that of the developing human brain – has been not been determined.

Here, we combined bulk tissue RNA-seq and single-cell RNA-seq to deeply profile lncRNA expression during neocortical development. Both polyA selected and total RNA were sequenced using strand-specific methods to comprehensively annotate and quantify lncRNAs in tissues. By applying this reference transcriptome to single-cell RNA-seq, we found that many lncRNAs are specific to distinct cell types and are abundantly expressed in individual cells. Furthermore, we found that the cell type-specific expression of lncRNAs contributes to the low levels of lncRNAs observed in tissues. Finally, using CRISPRi (clustered regularly interspaced short palindromic repeats interference) knockdown, we demonstrated that *LOC646329*, a lncRNA that appears low in neocortical tissues but high in the radial glia subpopulation, regulates cell proliferation.

## Results

### Catalogue of long non-coding RNAs in human neocortex development

**Fig. 1:**
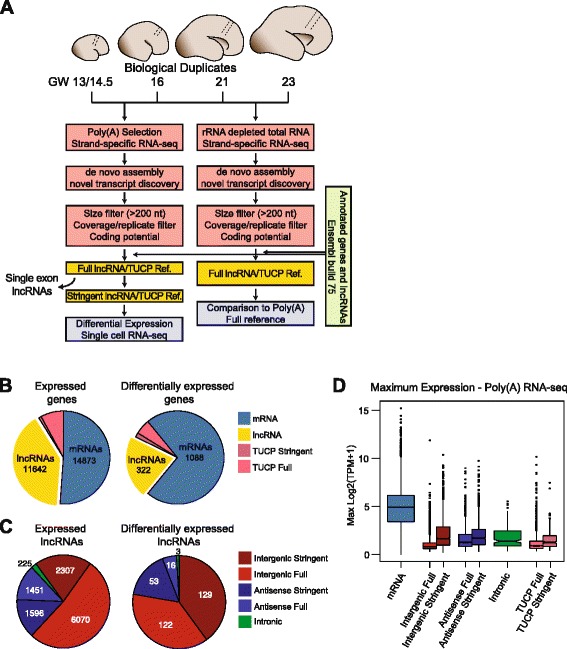
Catalogue of lncRNAs in human neocortex development. **a** Schematic of neocortex tissue dissection, poly(A) and total RNA-seq library prep, and computational pipeline for lncRNA annotation and quantification. **b** Numbers of expressed (*left*) and differentially expressed (*right*; DESeq2, FDR <0.01) mRNAs, lncRNAs, and TUCPs during neocortex development in bulk tissues. Stringent references omit novel single exon transcripts. **c** Breakdown of expressed (*left*) and differentially expressed (*right*) lncRNAs based on genomic orientation relative to mRNAs. **d** Maximum expression levels of transcripts described in the Full and Stringent references derived from Poly(A) selection RNA-seq, across all samples. TPM, Transcripts per Million

To identify lncRNAs expressed during human neocortical development, we microdissected radial sections of the tissue at gestational weeks (GW) 13/14.5, 16, 21, and 23. For each time point, we obtained biological duplicates and performed strand-specific RNA-seq of both polyA selected RNA and total RNA that had been rRNA depleted, generating over 200 million 100 bp paired-end mapped reads from each tissue specimen (Fig. 1a, Additional file 1: Table S1). After de novo transcriptome assembly of the polyA selected RNA-seq reads, previously annotated genes and short transcripts (<200 nt) were filtered. Transcript models that did not pass an optimized read coverage threshold in both biological replicates were removed (“Methods,” Additional file 2: Figure S1A). We then analyzed the protein coding potential of the remaining transcripts using three computational tools: CPC, CPAT, and Pfam [22–24], and any transcripts assigned a protein coding status by any of the three methods were classified as transcripts of uncertain coding potential (TUCP) [4] (Additional file 2: Figure S1B). These newly annotated transcripts were classified as intergenic, antisense, or intronic according to previously proposed nomenclature standards [25] and merged with the Ensembl build 75 transcriptome, resulting in the Full transcriptome reference. A Stringent transcriptome reference, in which novel single-exon transcripts were removed, was also generated (all deposited in GSE71315).

In our polyA selected reference transcriptomes, we identified 11,642 lncRNAs (4124 multi-exonic) and 2571 TUCPs expressed in developing human neocortex (Fig. 1b; Additional file 3: Table S2). The majority of lncRNAs were intergenic, though strand-specific RNA-seq enabled identification of 3047 antisense lncRNAs (Fig. 1c). A total of 8180 lncRNAs were novel to Ensembl 75/GENCODE v19 (Additional file 4: Table S3). Total of 7492, 7892, and 2105 were novel to the annotations of Cabili et al. [4], Hangauer et al. [7], and Mitranscriptome [6], respectively (Additional file 2: Figure S1C). On average, lncRNAs were detected at levels 13.6-fold lower than mRNAs (Fig. 1d). Novel polyA transcripts annotated from human brain tissues had genomic characteristics and conservation scores similar to previously annotated lncRNAs (Additional file 5: Figure S2).

**Fig. 2:**
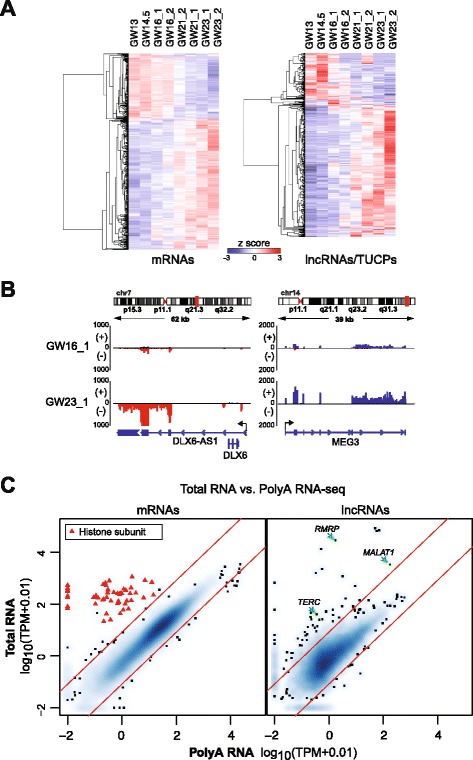
Differential expression of mRNAs and lncRNAs/TUCPs during neocortex development. **a** Heatmaps of differentially expressed mRNAs (*left*) and lncRNAs/TUCPs (*right*) throughout eight samples of bulk neocortex tissues. **b** Strand-specific RNA-seq alignments at the *DLX6-AS1* and *MEG3* loci in GW16 and GW23 replicate one sample*.* Scale, number of aligned reads. **c** Comparison of mRNA (*left*) and lncRNA (*right*) expression levels between poly(A) RNA-seq and total RNA-seq in GW16 sample 2. *Red diagonals* represent 10-fold enrichment in either total (*upper*) or polyA (*lower*) fractions. *Red triangles*, histone subunits enriched >10-fold in total RNA. TPM, Transcripts per Million

We next performed pairwise whole-transcriptome comparisons of all time points using DESeq2 [26] (FDR <0.01). A total of 1088 mRNAs and 424 polyA lncRNAs/TUCPs were differentially expressed across these time points (Fig. 2a, Additional file 6: Table S4). Among differentially expressed mRNAs, *PAX6* and *CENPA* were elevated in GW13-16, suggesting the increased presence of radial glia stem cells [27]. Conversely, *CUX2* and *ADCY1* were elevated in GW21-23, consistent with increased neurogenesis at these time points [28]. Among differentially expressed lncRNAs, *MEG3* and *DLX6-AS1* (a lncRNA antisense to the interneuron transcription factor *DLX6*), increased with developmental progression (Fig. 2b). Furthermore, gene ontology (GO) analysis of lncRNA gene neighbors suggested their function in neuronal differentiation (Additional file 2: Figure S1F).

Some lncRNAs, such as *MALAT1*, have been described to be non-polyadenylated [29]. To expand our catalogue to include non-polyA lncRNAs, we performed *de novo* transcriptome assembly with sequencing data from the total RNA (rRNA depleted) from each tissue sample. Full and Stringent lncRNA/TUCP references were generated with the same pipeline used for polyA selected transcripts (Fig. 1a). A total of 26,241 lncRNAs (4477 multi-exonic) and 4606 TUCPs were annotated from the total RNA-seq libraries (Additional file 2: Figure S1E). To identify transcripts that are likely to be non-polyA, we analyzed genes that were consistently >10-fold enriched in the total RNA libraries versus the polyA libraries across all samples (Fig. 2c, Additional file 7: Figure S3, and Additional file 8: Table S5). mRNAs that encode specific histone subunits are known to be non-polyA [30], and 52 out of the 58 mRNAs enriched in the total RNA-seq transcriptomes were for histone subunits, including *HIST1H2BK* and *HIST2H2AB*. By these methods, 85 lncRNAs were identified as non-polyA, with 65 being novel to Ensembl. Among the previously annotated lncRNAs in this set, known non-polyadenylated lncRNAs such as *MALAT1*, *RMRP*, and *TERC*, the RNA component of telomerase, were identified [31]. Thus, our transcriptome references allow broad profiling of lncRNAs during brain development, regardless of genomic orientation or polyadenylation status.

### Single-cell RNA-seq analysis of lncRNA expression

**Fig. 3:**
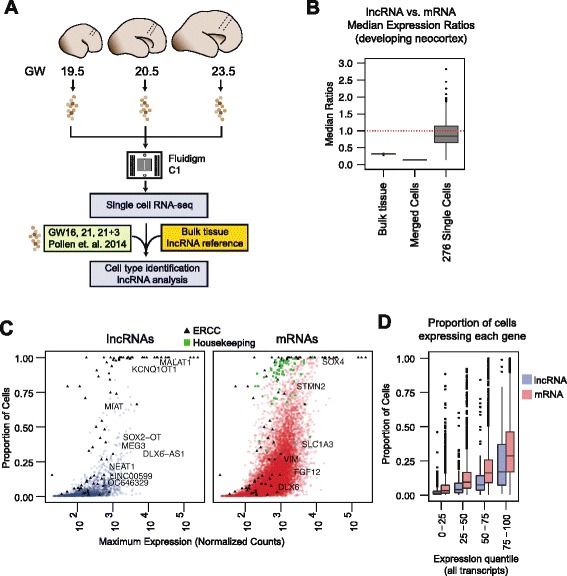
Single-cell transcriptomics of lncRNA expression. **a** Schematic of single-cell microfluidic capture and integration of transcriptome reference generated from bulk tissue RNA-seq to conduct cell-type identification and lncRNA analysis. Previously captured cells from Pollen et al. [32] were also included. **b** Distributions of median lncRNA expression to median mRNA expression ratios (lncRNA:mRNA) in bulk tissues, *in silico* merged single cells, and single cells from the developing neocortex. **c** Proportion of neocortex cells that expressed each lncRNA (*blue*) and mRNA (*red*), separated by maximum expression in single cells. **d** Same as in (**c**) but grouped by maximum expression quantile of the set of all transcripts (lncRNA and mRNA combined). *Green squares*, housekeeping genes; *black triangles*, ERCC Spike-In Controls

RNA-seq of whole tissues averages gene expression signatures of many different cell types [20]. To study lncRNA expression at single-cell resolution, we captured single cells from radial sections of GW19.5, GW20.5, and GW23.5 neocortex (Fig. 3a). To mitigate the effects of technical noise, we added equal amounts of ERCC Spike-In Control RNA to each single-cell lysis reaction. PolyA libraries were generated and a median of 1 million mapped read pairs were obtained for each reaction (Additional file 1: Table S1). We utilized our polyA Stringent transcriptome reference to perform transcriptome-guided genomic alignment and gene-level quantification of single-cell RNA-seq reads (Methods). Cells in which we detected >1000 genes and >40 ERCC species were retained, resulting in 226 single cells for consideration. We also included 50 single-cell libraries from GW16 and GW21 that we previously sequenced [32] (Additional file 9: Figure S4A-C). Although these libraries did not contain ERCC Spike-In Controls, they expanded the developmental range of our analyses.

To determine the sensitivity of our single-cell sequencing, we calculated the detection rate of each ERCC species (Additional file 9: Figure S4E). With the exception of ERCC-00116, which is inefficiently sampled by polyA selection [33], at more than eight copies, ERCCs were detected in 99–100 % of cells. We then fit a linear regression model relating normalized read counts (ncounts) to ERCC molecules across all cells (Additional file 9: Figure S4F). For our analyses, we included genes whose non-zero mean levels were above 20.6 ncounts, corresponding to two copies per cell. Furthermore, we omitted genes that were detected in fewer than three cells unless there was evidence of expression in our bulk RNA-seq data. Using these methods, we detected 10,929 mRNAs and 1400 lncRNAs expressed across the 276 cells (Additional file 10: Table S6).

### Abundant lncRNA expression in subpopulations of single cells

In tissue specimens, lncRNAs were detected at levels 13.6-fold lower than mRNAs on average, consistent with previous reports [4, 5, 7] (Fig. 1d). To determine whether lncRNAs may be expressed at high levels by subpopulations of cells, we analyzed the abundance of lncRNAs by comparing the median expression of lncRNAs to the median expression of mRNAs in each single cell (lncRNA:mRNA median ratios). In single cells, the median lncRNA:mRNA ratio was 0.85, with 32.2 % of cells exceeding 1.0 (Fig. 3b; Additional file 11: Figure S5A, F). To investigate whether the lncRNAs analyzed in the single cells exhibit lower expression in whole tissues, we analyzed the same set of lncRNAs and mRNAs in whole neocortical samples. In bulk tissue samples, the lncRNA:mRNA ratios were significantly lower compared to single-cell samples (median 0.31, *p* = 1.9 × 10^–6^, Mann–Whitney U; Fig. 3b, Additional file 11: Figure S5B). Furthermore, we merged the single cells in silico and found that this “reconstituted” sample had a lncRNA:mRNA ratio as low as the bulk tissues’ (ratio = 0.14, Fig. 3b; Additional file 11: Figure S5C). Analyses of cultured non-neural cells processed using the same single-cell isolation, library preparation, and computational pipeline provided additional evidence that the higher lncRNA:mRNA ratios observed in single neocortex cells is not primarily driven by the methods used (Additional file 12: Figure S6). Specifically, the more homogenous K562 cell line exhibited single-cell lncRNA:mRNA ratios (median 0.46) that were much lower than those from neocortex cells (Additional file 12: Figure S6A, D). Thus, lncRNAs that are detected at low levels in whole tissues can be abundantly expressed in individual cells.

To account for the observation that lncRNAs can be abundant in individual cells but detected at low levels in the whole tissues, we hypothesized that specific lncRNAs are expressed in subpopulations of cells. Housekeeping genes were detected in the vast majority of the single neocortex cells (median 91 %), as expected (Fig. 3c). In contrast, across all quantiles of gene expression, lncRNAs were detected in smaller proportions of cells than mRNAs (*p* = 2.7 × 10^–288^, Mann–Whitney U; Fig. 3c, d). Consistent with this observation, lncRNAs that were lowly detected in bulk tissues were also expressed in fewer single cells (Additional file 11: Figure S5D), indicating that in heterogeneous tissues lncRNAs are expressed in more discrete populations of cells than mRNAs.

### Cell type-specific expression of lncRNAs

**Fig. 4:**
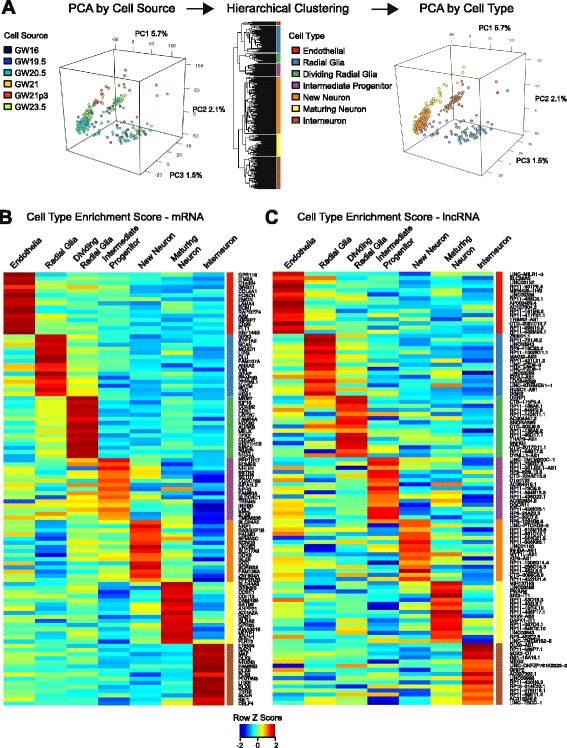
Cell type-specific expression of lncRNAs. **a** Identifying cell types using unsupervised clustering. *Left* – Principal component analysis (PCA) of single cells colored by developmental stage of source tissues. *Middle* – Complete linkage hierarchical clustering of single cells using genes exhibiting variance greater than expected than from technical noise. *Right* – PCA of single cells colored by cell types inferred from protein coding genes specific to each cluster. Axes labels indicate percent variation explained by each PC. **b** Heatmaps of cell type enrichment scores for the 15 most specific mRNAs and (**c**) lncRNAs in each cluster. GW21p3, primary cells derived from GW21 brain that were cultured in differentiation media for 3 days

To determine whether the discrete and abundant expression of lncRNAs could relate to their expression in molecularly distinct cell types of the developing human brain, we performed hierarchical clustering of single cells with genes that exhibited significant variability (Methods, Fig. 4a, Additional file 9: Figure S4D, and Additional file 13: Figure S7). We identified seven clusters, each comprising cells derived from at least three different brains (Additional file 13: Figure S7). To infer the identity of these cell clusters, we determined the most specific mRNAs in each cluster (Methods, Fig. 4b, Additional file 14: Table S7) and compared these genes to known cell type-specific markers. With these methods, we identified the cell clusters to be endothelial cells (*FLT1*), radial glia (*VIM*, *GFAP*), dividing radial glia (*MKI67*, *TOP2A*), intermediate progenitors (*EOMES*), newborn neurons (*SEMA3C*, *DCC*), maturing excitatory neurons (*SATB2*, *ADCY1*), and inhibitory interneurons (*DLX2*, *GAD1*) [28, 34–36].

To identify cell type-specific lncRNAs, we ranked the most specific lncRNAs of each cluster (Fig. 4c). Overall, lncRNAs exhibited specificity scores comparable to those of mRNAs, with lower abundance lncRNAs having slightly greater specificity than abundance-matched mRNAs (*p* = 0.01; Mann–Whitney U; Additional file 15: Figure S8C). Notably, lncRNAs that were detected at lower abundances in bulk tissues were more cell type-specific in single cells than higher abundance lncRNAs (*p* = 1.1 × 10^–47^, Mann–Whitney U; Additional file 15: Figure S8D). Of the top 105 specific lncRNAs (15 in each of seven clusters), 10 were not annotated in Ensembl. *DLX6-AS1*, whose mean expression was 6123-fold higher in interneurons than in all other cell types, exhibited the highest cell type-specific enrichment of any gene (Fig. 4c). Its mouse ortholog *Evf2* has been shown to function in interneurons [3, 37]. While *MEG3* and *SOX2-OT* have been shown as brain- and even neuron-specific [34], our clustering revealed these lncRNAs to be more specific to interneurons than to newborn or maturing excitatory neurons (Fig. 4c).

Gene co-expression analyses have previously been used to infer biological functions for novel lncRNAs [5, 38]. We therefore constructed co-expression networks between the top specific lncRNAs and all mRNAs expressed in the single cells (Additional file 16: Figure S9A). Isolating the top 10 % most correlated or anticorrelated mRNAs to these lncRNAs revealed gene clusters with cell type-specific function, such as “angiogenesis” for the endothelial lncRNAs and “GABA synthesis, release, reuptake and degradation” for the interneuron lncRNAs (Additional file 16: Figure S9B).

**Fig. 5:**
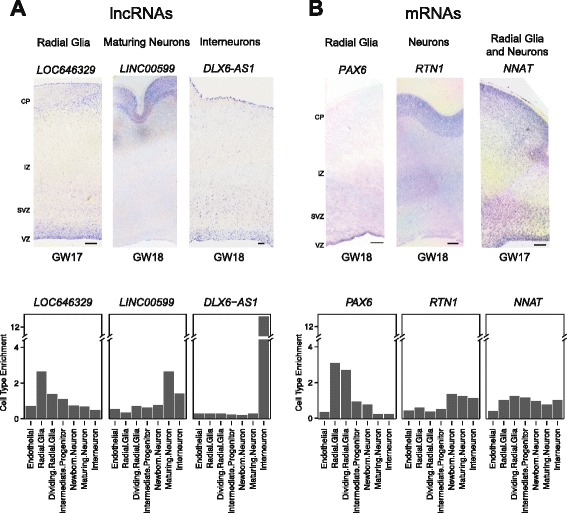
*In situ* hybridization of cell type-specific lncRNAs and mRNAs in developing neocortex. **a** *In situ* hybridizations and corresponding cell type enrichment values for radial glia-specific lncRNA *LOC646329* (*left*), maturing neuron-specific lncRNA *LINC00599* (*middle*), and interneuron-specific lncRNA *DLX6-AS1* (*right*). **b** *In situ* hybridizations and corresponding cell type enrichment values for radial glia-specific mRNA *PAX6* (*left*), neuron mRNA marker *RTN1* (*middle*), and progenitor and differentiated cell-expressed mRNA *NNAT* (*right*). Scale bars, 250 μm. CP, cortical plate. IZ, intermediate zone. SVZ, subventricular zone. VZ, ventricular zone

To validate our cell type-specific lncRNA expression patterns, we performed *in situ* hybridizations for three lncRNAs: *LOC646329* (radial glia), *LINC00599* (maturing neuron), and *DLX6-AS1* (interneuron) (Fig. 5a). *LOC646329* was enriched in the ventricular zone (VZ), where most radial glia reside. *LINC00599* was enriched in the cortical plate (CP), which harbors maturing neurons. *DLX6-AS1* was enriched in the subpial granular layer and also exhibited a gradient of punctate expression spanning from the VZ to the intermediate zone (IZ), consistent with the migration patterns of cortical interneurons [39, 40]. Imaging of the radial glial marker *PAX6*, the neuron marker *RTN1*, the maturing neuron marker ADRA2A, and the control marker *NNAT*, which is expressed broadly across progenitor and differentiated cells [32], further validated the regional expression patterns of the cell type-specific lncRNAs (Fig. 5b, Additional file 17: Figure S10).

**Fig. 6:**
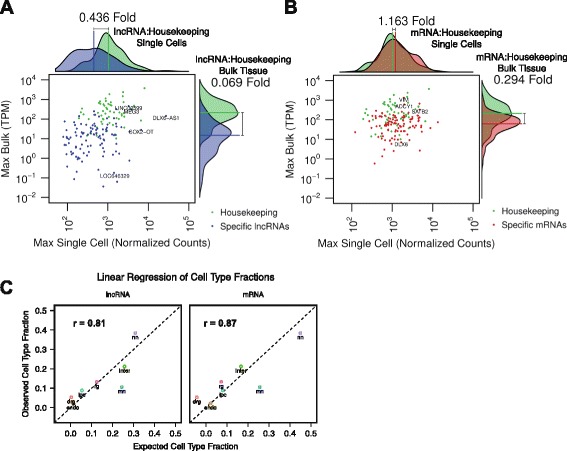
Cell type-specific lncRNAs appear to be lowly expressed in bulk tissues. **a** Comparison of single-cell and bulk tissue maximum expression levels of 105 cell type-specific lncRNAs and (**b**) 105 cell type-specific mRNAs. *Green*, housekeeping genes; *blue*, cell type-specific lncRNAs; *red*, cell type-specific mRNAs. Projected density plots summarize expression levels of scatterplots along the single-cell (*horizontal*) and bulk tissue (*vertical*) axes. Fold changes noted alongside the projected density plots represent the ratio of the median expression of cell type-specific lncRNAs or mRNAs to the median expression of housekeeping genes in single cell or whole tissue RNA-seq. **c** Comparison of expected cell type fractions as predicted by linear regression (*x axis*) and observed cell type fractions (*y axis*). TPM, Transcripts per million; Endo, endothelial; rg, radial glia; drg, dividing radial glia; ipc, intermediate progenitor cell; nn, newborn neurons; mn, maturing neurons; inter, interneurons

To ask whether cell type-specific expression contributes to genes being detected at low levels in tissues, we analyzed the expression levels of the top 105 cell-type specific mRNAs and lncRNAs. As expected, in bulk tissues, cell type-specific mRNAs were detected at lower levels as compared to housekeeping genes (Fig. 6b). Cell type-specific lncRNAs were detected at even lower abundances than housekeeping genes (0.069-fold). In contrast, in single cells, these lncRNAs were detected at levels close to those of housekeeping genes (0.436-fold, Fig. 6a, Additional file 15: Figure S8A, B). Thus, these cell type-specific lncRNAs appeared 6.31 times less abundant in bulk tissues as compared to their expression in single cells.

We then reasoned that the tissue level expression of a given gene could be modeled as a weighted average of cell type-specific expression, where the weights are proportional to the relative abundance of each cell type. We used multiple linear regression to estimate the expected fraction of each cell type, using the mean expression in each cell type as predictor variables and bulk expression as the response. For consistency, only single cells derived from all cortical layers between GW19.5 and 23.5 were considered, and only bulk tissues from GW21–23 were used. Remarkably, the expected fractions of cell types showed strong agreement with the observed cell types identified in this study (Fig. 6c). This was true for models using lncRNAs only (r = 0.81) and mRNAs only (r = 0.87), showing that the degree of decreased expression in bulk tissues can be explained largely by the relative abundances of cell types. Nonetheless, the discreteness of lncRNA expression (Fig. 3d), and the relatively lower explanatory power of the lncRNA linear model (R^2^ = 0.336; mRNA R^2^ = 0.422) suggest that lncRNAs exhibit additional expression variation even within cell types.

### Radial glia-enriched lncRNA *LOC646329* regulates cell proliferation

**Fig. 7:**
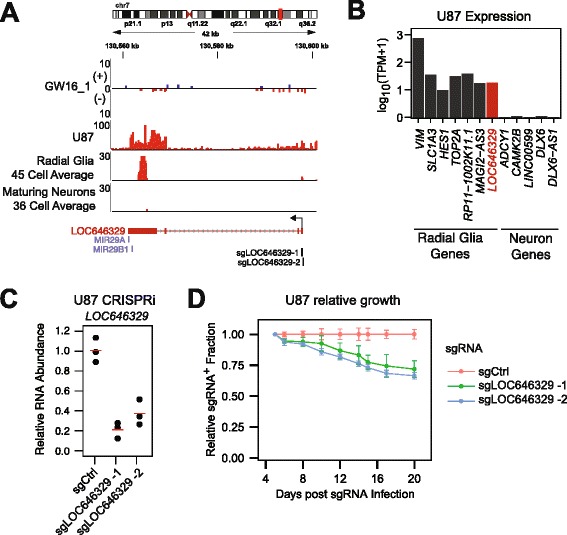
CRISPRi knockdown of radial glia-enriched lncRNA *LOC646329* inhibits proliferation. **a** RNA-seq alignments at the *LOC646329* locus. GW16(+)/(–) replicate one and U87 alignments are number of reads. Radial glia and maturing neurons are merged alignments normalized by number of cells within each cell cluster. sgRNAs targeting the TSS of *LOC646329* are indicated. **b** Expression of radial glia and neuron mRNAs and lncRNAs in U87 glioblastoma cells. **c** qPCR of *LOC646329* following 4 days of CRISPRi knockdown using two sgRNAs targeting the TSS of *LOC646329* relative to non-targeting control sgRNA. Biological triplicates (*black circles*) show 79.0 % repression with sgLOC646329-1 (*p* = 0.0014; Welch’s t-test) and 62.6 % repression in sgLOC646329-2 (*p* = 0.0035; Welch’s t-test). *Red lines*, mean. **d** Relative growth assays of U87 cells following sgRNA infection. sgRNA^+^ fraction was calculated relative to 5 days post sgRNA infection and normalized to the sgCtrl^+^ fraction at each time point. Biological triplicates show 28.1 % depletion at 20 days with sgLOC646329-1 (*p* = 0.0073; Welch’s t-test) and 33.5 % depletion with sgLOC646329-2 (*p* = 0.00048; Welch’s t-test). Error bars, standard deviation of triplicate cultures

*LOC646329* was among the most radial glia-enriched lncRNAs, and it was also detected at very low levels in bulk tissues (Transcripts per Million (TPM) <0.5, Figs. 4c and 7a). Radial glia – the neural stem cell population of the developing brain – share biological and transcriptional characteristics with glioblastoma multiforme (GBM), a malignant glial tumor (Fig. 7b) [41]. *LOC646329* is expressed in human GBM, including the U87 GBM cell line (Fig. 7a, b). To investigate the biological function of *LOC646329*, we performed CRISPRi knockdown of the lncRNA in U87 cells. U87 cells stably expressing dCas9-KRAB were infected with one of two lentiviruses harboring distinct sgRNAs targeting the transcription start site of *LOC646329* (Fig. 7a). After confirming knockdown of *LOC646329* by qPCR, we performed internally controlled growth assays by measuring the percentage of cells infected with sgRNA-expressing lentivirus over time (Fig. 7c, d). Knockdown of *LOC646329* with either sgRNA reduced the propagation of U87 cells, indicating an important role for this lncRNA in cell proliferation.

## Discussion

LncRNAs are remarkably tissue specific [1, 4, 5] and the mammalian brain expresses a tremendous diversity and number of this class of non-coding transcripts [4, 9]. Furthermore, some neural lncRNAs are primate and/or human specific, suggesting that lncRNAs play a role in the evolutionary expansion of the human neocortex [42, 43]. To lay groundwork for the study of lncRNAs in human brain development and disease, we generated a reference catalogue of lncRNAs expressed at different stages of human neocortex development.

Our human neocortex lncRNA reference catalogue greatly improves upon previous annotations of human neural lncRNAs in several ways [15, 43]. First, we studied several developmental stages (eight samples spanning GW13 to GW23). Second, we analyzed both polyA selected and total RNA from each of the tissue specimens, which enabled the identification of novel lncRNAs that are potentially non-polyadenylated. Third, all of our cDNA libraries retained strand-of-origin information, allowing more accurate annotation and quantification of antisense transcripts, several of which have been shown to have important functions [17–19]. Finally, the inclusion of biological replicate samples at matched (or near matched) developmental stages also allowed us to identify lncRNAs that were differentially expressed over time. We anticipate that this reference will facilitate future studies focusing on the roles of lncRNAs in cortical development and evolution, including cell type-specific roles of individual lncRNAs.

Our single-cell transcriptome analyses indicate that lncRNAs can be highly expressed in individual cells of the developing neocortex. In cultured cells, studies using single molecule fluorescence in situ hybridization (FISH) demonstrate relatively uniform expression of 61 different lncRNAs across all cells, and that cells expressing high levels of specific lncRNAs are uncommon [21]. Consistent with these observations in cell lines, we found that lncRNA abundance is uniformly low to moderate in single cells of the relatively homogenous K562 leukemia line (Additional file 12: Figure S6A, D). In contrast, single cells from developing human neocortex exhibited a wide range of lncRNA abundances, with some lncRNAs such as *SOX2-OT* and *DLX6-AS1* reaching levels higher than those of housekeeping genes (Fig. 6a, Additional file 15: Figure S8B). Given that the cultured K562 and human neocortex cells were all processed using the same Fluidigm C_1_ platform and processed with the same computational pipeline, the discrete and abundant expression of lncRNAs in neocortical cells likely relates to differences in their cellular identity.

Consistent with this hypothesis of cellular identity explaining lncRNA expression, our results indicate preferential “dilution” of lncRNAs in bulk samples (Fig. 6a), suggesting that cell type-specific expression of lncRNAs contributes to the apparent low expression of lncRNAs in heterogenous tissues. Furthermore, we were able to predict the relative abundances of the cell types identified in this study by regressing bulk expression onto cell type-specific expression (Fig. 6c). However, two caveats are that we have not captured all the cell types in the developing human cortex, and we have not ruled out the possibility that the processing of single cells may enrich for certain cell types. A much larger scale study, such as those performed on the adult mouse brain [44] and retina [45], would be required to better understand relative abundances of cell types in the developing human brain.

Quantitative measurements in single-cell RNA-seq have been refined by the use of unique molecular identifiers (UMIs) that deconvolute non-uniform amplification of cDNA [44–47]. However, these methods are not yet compatible with full-length transcript coverage, which is advantageous for the study of lncRNAs. We retained full-length transcript coverage using the SMARTer protocol and instead used ERCC Spike-In Control RNA to control for technical noise. Our use of these synthetic RNA spikes to determine an expression threshold and to identify variable genes greatly diminished technical noise as the primary driver of lncRNA abundance. However, the use of spike-ins set a lower bound of detection and many truly low abundance lncRNAs likely fell below this threshold. Nonetheless, the same threshold was also used for mRNAs, and our internal comparisons of lncRNAs to mRNAs in the same cell also controls for technical noise, especially since lncRNAs are not necessarily less stable than mRNAs [48]. Therefore, our observation of discrete and abundant expression of lncRNAs in individual cells is not primarily driven by technical noise.

Several lncRNAs have previously been described as brain specific [4, 12, 43]. In this study, we found that these as well as novel lncRNAs can be further attributed to distinct cell types within the neocortex. For instance, *MEG3* is highly expressed in the brain [4, 49] and we found this lncRNA to be especially enriched in interneurons, with moderate expression in maturing excitatory neurons (Fig. 4c). We also identified cell type-specific and cortical layer-specific expression of lncRNAs such as *LINC00599 and LOC646329* (Fig. 5), which have not been studied at this resolution in the human brain. In addition, our study of cell type-specific transcripts revealed a role for *LOC646329* in regulating cell proliferation. Determining whether *LOC646329* acts as a host to the microRNAs *MIR29A/B1* (Fig. 7a) or through a distinct and separable role [50] will require further investigation. Nonetheless, this result illustrates that lncRNAs that appear to be lowly expressed in tissues can have important functions and motivates the study of lncRNAs at the single-cell level.

## Conclusions

Whole transcriptome analysis of single cells, in combination with deep RNA-seq of tissues, allowed us to identify abundant cell type-specific lncRNAs in the developing neocortex. Importantly, many lncRNAs that appear lowly expressed at the population level are abundant in discrete cell types. Analysis of these lncRNAs in the brain both reveals useful markers of cell types during lineage progression from precursor to differentiated cell types and enables the discovery of novel cellular functions of lncRNAs. As such, lncRNAs that are lowly expressed in a population may still regulate essential functions and should not be discounted solely based on apparent abundance. These data and workflow should facilitate future studies aimed at determining the function of lncRNAs in the brain during development and disease.

## Methods

### Prenatal tissue collection

De-identified human prenatal brain tissue samples were collected from elective pregnancy termination specimens, usually within 2 h of the procedure. Gestational age was determined by measuring foot length and tissues were transported on ice in Leibowitz-15 medium for immediate tissue processing and RNA extraction.

### Ethics

Research protocols were approved by the Human Gamete, Embryo, and Stem Cell Research (GESCR) Committee (Institutional Review Board) at University of California, San Francisco (10-03379). Donated specimens were examined only from patients who had previously given informed consent and in strict observance of state and institutional legal and ethical requirements. All experimental methods comply with the Declaration of Helsinki.

### Bulk tissue RNA-seq library preparation

Fresh neocortex tissues were dissected along the radial axis, and 50–100 mg sections spanning the ventricular zone to the marginal zone of the neocortex were harvested in TRIzol. Mixtures were homogenized by triturating 10 times through a 21G needle. Ethanol extracted RNA was loaded onto RNeasy columns (QIAGEN) and on-column DNase treatment was performed as previously described [5]. Purified RNA samples produced RIN scores between 8.6 and 9.5, measured by 2100 Bioanalyzer (Agilent). RNA-seq libraries were generated using both TruSeq Stranded mRNA and TruSeq Stranded Total RNA with Ribo-Zero Gold kits (Illumina) according to the manufacturer’s protocols. cDNA validation and normalization were performed using RT-PCR and Quant-iT PicoGreen (Invitrogen). Cluster generation and high-throughput sequencing were performed on a HiSeq 2500 (Illumina), using the paired-end 100 bp protocol.

### Single-cell capture and RNA-seq library preparation

Tissues were microdissected by embedding in 3.5 % low melt agarose (Fisher) and sectioned along the radial axis, perpendicular to the ventricles, using a Leica VT1200S vibratome in artificial cerebrospinal fluid (ACSF) media containing 125 mM NaCl, 2.5 mM KCl, 1 mM MgCl_2_, 1 mM CaCl_2_, and 1.25 mM NaH_2_PO_4_. Cortex dissections were added to papain (Worthington Biochem. Corp) and 2000 units/mL of DNase I freshly diluted in EBSS and incubated at 37 ° C for 30 min and centrifuged for 5 min at 300 g. Supernatants were removed and dissociated cells were resuspended in 0.5 mL of sterile DPBS containing 3 % FBS (Sigma) and 1000 units of DNAse I. Suspensions were further dissociated by pipetting up and down 10 times and then passing through a 40 μm strainer cap (BD Falcon) to yield single-cell suspensions. Single-cell dissociations were performed on samples separate from those used for bulk tissue RNA-seq.

Single-cell capture was performed using the Fluidigm C1 Single-Cell Auto Prep Integrated Fluidic Circuit (IFC) and SMARTer Ultra Low RNA Kit as previously described [32]. We minimized the capture of cell doublets by only using small (5–10 μm) 96-plex IFCs, which in combination with neural cells were demonstrated to have the lowest instance of doublets (median 6 %, SD 4 %) [51]. ERCC Spike-In Controls (Ambion) were added to each single-cell lysis reaction at a final dilution of 1:20,000, in order to capture a wide range of molecule count per reaction. cDNA was quantified using High Sensitivity DNA Kits (Agilent) and diluted to 0.15–0.30 ng/μL in C_1_ Harvest Reagent. Dual indexing and amplification were performed using the Nextera XT DNA Sample Preparation Kit (illumina) with the following modifications: reactions were performed at one-quarter of the recommended volume, tagmentation proceeded for 10 min, and the PCR extension time was 60 s. Amplified cDNA was size selected twice using 0.9X volume of Agencourt AMPure XP beads (Beckman Coulter). Final cDNA libraries were quantified using High Sensitivity DNA Kits (Agilent) and sequenced on a HiSeq 2500 (Illumina), using the paired-end 100 bp protocol.

### LncRNA identification and quantification pipeline – bulk whole tissues alignment and de novo transcript assembly

Quality control of RNA-seq reads was performed using FastQC. No read trimming was performed on reads from bulk tissues, since all samples exhibited 25%ile quality scores above Q30 at all 100 bp positions. Strand-specific reads were aligned to the human reference genome, Ensembl GRCh37/hg19 release 75, using TopHat v2.0.10 with the flags (--library-type fr-firststrand --microexon-search). Each sample generated between 105 and 148 million mapped read pairs from rRNA depleted total RNA-seq, and between 60 and 105 million mapped read pairs from polyA selection RNA-seq (Additional file 1: Table S1). De novo transcriptome assembly was performed separately on rRNA depletion total RNA-seq alignments, and on polyA selection RNA-seq alignments, using Cufflinks v2.2.1 with the flags (-M ensembl_75_mtRNA_rRNA.gtf -b genome.fa -u --library-type fr-firststrand --max-multiread-fraction 0.25 --3-overhang-tolerance 2000) to mask potential rRNA and mtRNA reads, enable bias correction and multi-map correction, and also to reduce the identification of polymerase run-on fragments as novel transcripts [52]. Transcriptome assemblies at all developmental stages and replicates were merged, separately for rRNA depletion total RNA-seq and polyA selection RNA-seq, with the Ensembl 75/GENCODE 19 reference transcriptome, using Cuffmerge. To identify transcripts novel as compared with Ensembl, we utilized Cuffcompare class codes and extracted those assembled transcripts classified as: i – novel intronic, u – novel intergenic, x – novel antisense. All novel transcripts under 200 nt in length were removed. Of the remaining transcripts, we determined minimal read coverage thresholds based on whether Cufflinks classified previously annotated transcripts as having “full_read_support.” By analyzing the true positive rate versus false positive rate of classifying known genes as obtaining “full_read_support” at various coverage thresholds, we determined the minimum coverage to be 1.4 for polyA and 1.67 for total RNA-seq (at FDR = 0.05).

Starting with just the polyA RNA-seq data, transcripts with read coverage above 1.4 in both biological replicates of at least one developmental stage were included in the reference and considered to be expressed in the neocortex. Due to limited availability of early fetal tissue, the GW14.5 sample was treated as the biological duplicate of the GW13 sample. Novel transcripts that were predicted to have protein coding capability by one or more of the following methods were classified as transcripts of uncertain coding potential (TUCP): CPAT [23], threshold = 0.364; CPC [22], threshold = 0; Pfam [24]. For comparing to the Pfam database, the longest potential open reading frame (ORF) of each novel transcript was obtained and any putative ORF that had a significant match for a protein domain annotated in Pfam A or Pfam B resulted in the parent transcript being classified as a TUCP. All remaining novel lncRNAs and TUCPs were then named according to recently proposed nomenclature standards [25], for instance LINC-[nearest mRNA] for intergenic lncRNAs and [nearest mRNA]-AS for antisense lncRNAs, and were then merged to the Ensembl 75 reference transcriptome, resulting in the polyA Full reference transcriptome. The polyA Stringent reference transcriptome was produced by removing all novel single-exon lncRNAs and TUCPs. Known lncRNAs from Ensembl were obtained by identifying transcripts with one of the following biotype classifications: “3prime_overlapping_ncrna,” “antisense,” “lincRNA,” “processed_transcript,” “sense_intronic,” and “sense_overlapping.” The same pipeline, with the coverage threshold of 1.67, was performed for reads derived from the total RNA-seq.

### LncRNA quantification

Gene-level fragment counts for each polyA and total RNA sample were quantified using featureCounts v1.4.6 [53], using the flags: -p -s 2 -B -C -t exon -g gene_id. Count tables were normalized to Transcripts per Million [54] for internal comparisons and visualizations of bulk RNA-seq. To identify differentially expressed genes, we used DESeq2 [26] on gene-level fragment counts derived from the polyA samples and polyA Full reference transcriptome. Pairwise negative binomial significance tests were performed between developmental stages using biological duplicates and the union of genes that were significant at FDR <0.01 were classified as differentially expressed. Gene ontology (GO) analysis was performed using the DAVID web server [55]. To identify lncRNAs enriched in total RNA versus polyA RNA, we first identified all transcript annotations in the total Full reference that did not overlap with transcripts in the polyA Full reference. We then merged these transcripts with the polyA Full reference. We quantified gene-level fragment counts annotated in this augmented reference using all samples (both polyA RNA and total RNA), as described above. For each of the eight tissue samples, we then compared the TPM for each gene as observed from the polyA and total RNA-seq. mRNAs and lncRNAs that were consistently >10-fold enriched in one or the other fraction across all eight samples were then considered enriched in total RNA or enriched in polyA RNA.

### Quality control and analysis of single-cell RNA-seq

Paired end 100 reads from single-cell cDNA libraries were quality trimmed using Trim Galore with the flags: -q 20 --nextera --length 20. Trimmed reads were aligned to the human reference genome, Ensembl GRCh37/hg19 release 75, augmented with the 92 ERCC Spike-In Control sequences, using TopHat v2.0.10 with the flags: --transcriptome-index = polya_stringent_reference.gtf --prefilter-multihits. The polyA Stringent reference transcriptome, derived from bulk tissue RNA-seq as described above, was used as a transcriptome reference. A median of 1 million 100 bp paired-end reads were successfully aligned per cell. Gene-level fragment counts were quantified using featureCounts v1.4.6 with the flags: -p -B -C -t exon -g gene_id. Since the SMARTer cDNA prep does not retain strand-of-origin information, we did not count reads that overlapped multiple features, even if they were annotated on opposite strands. Outlier identification was performed by calculating the number of genes and ERCC species detected in each cell (defined as the number of genes with >1 count). The distribution of genes and ERCCs detected decayed rapidly below 1000 genes and 40 spikes, with the remaining few samples centered at 0 genes and 0 spikes (Additional file 9: Figure S4A, B). Therefore, we excluded from analyses cells in which we detected fewer than 1000 genes and 40 ERCC spikes. The >40 ERCC requirement was not used for previously sequenced single cells from Pollen et al. [32], as these cells from GW16 and GW21 brains did not contain ERCC Spike-In Controls.

To determine the most accurate expression metric to use for our single-cell transcriptome analyses, we correlated absolute ERCC spike abundances in each cell with their expression readout using four different metrics (Additional file 9: Figure S4J). Count-based metrics, which were counts and CPM (Counts per Million mapped reads), outperformed length-normalized metrics, which were FPKM (Fragments per Kilobase per Million mapped reads) and TPM (Transcripts per Million). Therefore, we used counts and normalized each single-cell library by transcriptome size factors according to DESeq [26]. Separate size factors were calculated and used for genes and ERCCs.

Detection rate analysis was performed by calculating for each gene and ERCC spike the number of cells out of the 276 neocortex cells that exhibited >1 normalized counts for that gene. We determined that the sensitivity of detection was 74 % for a ERCC species present at two copies per cell. At more than eight copies per cell, ERCCs were detected in 99–100 % of cells (Additional file 9: Figure S4E). The exception was ERCC-00116, which is known to be inefficiently sampled by polyA selection [33]. To find a reliable count threshold for further analyses, we then fit a linear regression model relating normalized read counts (ncounts) to ERCC molecules across all cells (Additional file 9: Figure S4F). We then included genes whose non-zero mean levels across all cells were above 20.6 ncounts, corresponding to two copies per cell. Furthermore, we omitted genes that were detected in fewer than three single cells unless they were considered to be expressed by the whole-tissue RNA-seq experiments. For single-cell comparisons of gene expression levels of lncRNAs to mRNAs, only genes that were expressed above two normalized counts in each cell were considered. The median normalized counts for lncRNAs was then compared to the median normalized counts for mRNAs in single cells. In silico merged cells in Fig. 3b and Additional file 11: Figure S5C were generated by taking the sum of gene read counts across all single cells that passed QC.

### Clustering and cell type identification of single cells

To identify genes for unsupervised clustering, we modeled technical noise using ERCC Spike-In Control RNA and identified genes with significantly greater expression variation than expected from noise according to Brennecke et al. [56]. Briefly, we fit a gamma family generalized linear model to the coefficient of variation squared of ERCCs as a function of mean normalized counts. We then determine the expected variance model for genes that exhibit greater than 50 % biological coefficient of variation at FDR <0.01 (Benjamini–Hochberg adjusted *p* value from Chi-square distribution). These 5243 remaining genes were then used for principal component analysis (PCA). PCA was performed using log_2_ size factor-normalized counts with a pseudocount of 1 and visualized in R. We then ranked all genes (mRNAs and lncRNAs) based on their highest absolute values of gene loading scores across the first four principal components in PCA. We then performed complete linkage hierarchical clustering of single-cell expression (log_2_(Normalized Counts + 1)) using 1-(Pearson correlation coefficients) as the distance metric, using the top 500 genes ranked by PCA loading scores (Additional file 13: Figure S7). Cluster dendrograms were cut statically between r = 0.8 and r = 0.9, and cell types were inferred by comparing mRNAs specific to each cluster to known markers.

### Cell type-specificity analysis

Cell type-specificity for each gene was calculated as the odds ratio of a cell expressing a given gene, above a given threshold, within a cluster compared to outside a cluster. Specifically, if *p*_*ij*_ is the fraction of cells expressing gene *i* above threshold in cluster *j*, and *q*_*ij*_ is the fraction of cells expressing gene *i* above threshold that are not in cluster *j*, then we measure cell type specific expression odds by: ${\theta}_{ij}= log\frac{p_{ij}\left(1-{q}_{ij}\right)}{q_{ij}\left(1-{p}_{ij}\right)}$. To generate the set of mRNAs and lncRNAs specific to each cell type, we first assigned each gene to a cell type by determining which cell type yielded the maximum log odds specificity score for that gene. We used each gene’s own 75%ile for expression thresholds. Then we calculated expression enrichment scores for each gene in each cell type by: *e*_*ij*_ = *u*_*ij*_/*v*_*ij*_, where *u*_*ij*_ is the mean expression level of gene *i* within cluster *j*, and *v*_*ij*_ is the mean expression level of gene *i* outside of cluster *j*. Genes were ranked within their cell type cluster assignments using expression enrichment scores and the top 15 mRNAs and lncRNAs in each cell type were obtained.

### Linear regression model

We reasoned that the relationship between bulk tissue RNA-seq expression and single-cell RNA-seq expression could be approximated using multiple linear regression in the form:

$$ Y = {\beta}_0+{\beta}_1{X}_1+{\beta}_2{X}_2+\dots +{\beta}_n{X}_n $$

where *Y* is bulk expression, *n* is the number of cell types identified in the study, *β*_0_ is a constant that normalizes global differences between single-cell and bulk RNA-seq, [*β*_1_ … *β*_*n*_] is the vector of slope coefficients that are proportional to the relative fraction of each cell type within the bulk tissue, and *X* is the vector of mean expression of each cell type as measured by single-cell RNA-seq. Expression values were represented as log_2_ transformed, size factor-normalized counts, with a pseudocount of 1. To improve matching between bulk tissues and single cells, we used the mean expression of GW21 and GW23 bulk cortex samples (n = 4) as the bulk expression level, and we used single cells derived from all cortical layers between GW19.5 and 23.5 (n = 226) and calculated the mean expression for each cell type, as was determined by hierarchical clustering in Fig. 4. Only genes that passed the single-cell expression threshold (described above), and were detected above 5 counts in bulk tissues, were included. The data were then fit using the *lm* function in R, and the resulting slope coefficients were normalized such that ∑_*i* = 1_^*n*^*β*_*i*_ = 1. These normalized coefficients, which represent the expected fractions of cell types, were then compared to the observed relative fractions of cell types, according to the cluster sizes in Fig. 4 (but only counting the 226 included cells).

### Gene co-expression analysis

A correlation matrix was assembled by calculating all pairwise Pearson correlation coefficients between the 105 cell type-specific lncRNAs and all expressed mRNAs across single cells. Expression values were represented as log_2_ transformed, size factor-normalized counts, with a pseudocount of 1. mRNAs whose maximum, absolute value correlation coefficients were in the top 10%ile were kept. The resulting sub-matrix was clustered using Euclidean distance and complete linkage, and gene clusters were analyzed for gene ontology terms using Enrichr [57]. Clusters with significant gene ontology terms are represented in Additional file 16: Figure S9.

### *In situ* hybridization

Probes for *in situ* hybridization were synthesized (Genscript) or cloned from GW16 human fetal neocortex cDNA, which were reverse-transcribed using SuperscriptII (Invitrogen) with random hexamer primers. Probe sequences were subcloned into pGEM T-Easy vector (Promega). T7 or SP6 RNA polymerase (Roche) was then used for in vitro transcription of the probes, in the presence of DIG-RNA Labeling Mix (Roche). *In situ* hybridization was performed blinded to the sense/antisense status for each probe and sense control probes gave no signal (data not shown). The in situ hybridization protocol has been described before [58]. Images were collected with a Leica DMI 4000B microscope using a Leica DFC295 camera.

### Immunohistochemistry

Immunohistochemistry was performed as described in Pollen et al. [32]. Briefly, tissue samples were fixed in 4 % paraformaldehyde, cryoprotected in 30 % sucrose, and embedded in a 1:1 mixture of 30 % sucrose and optimal cutting temperature (Thermo Scientific). Cryosections of 20 μm were collected using a Leica CM3050S cryostat. Primary antibody: ADRA2A (1:100, Thermo Scientific, PA1-048). Heat-induced antigen retrieval was performed in 10 mM sodium citrate buffer, pH 6. Binding was revealed using Alexa Fluor™ 488 fluorophore-conjugated secondary antibody (Life Technologies). Images were collected with a Leica TCS SP5 X Confocal microscope.

### CRISPRi knockdown of lncRNAs

CRISPRi mediated repression of lncRNA transcription was performed as described previously [59]. First, we created a stable polyclonal cell line expressing dCas9-KRAB by transducing U87 glioblastoma cells with dCas9-KRAB-BFP lentivirus and sorting for the top 30 % of BFP expressing cells. sgRNAs were designed as previously described [59]. sgRNA protospacer sequences were: sgLOC646329-1, GCTTAGGAAATCACCAGCTCC; sgLOC646329-2, GGTCTGCCGTGACAGTTCAGT; sgCtrl, GAACGACTAGTTAGGCGTGTA. We then cloned the sgRNA sequences into puromycin-resistant lentiviral vectors and infected dCas9-KRAB U87 cells with the resulting lentivirus particles. To assess lncRNA knockdown, we treated cells with 1 μg/mL puromycin for 4 days following sgRNA infection and performed RT-qPCR as previously described [59]. RT-qPCR primers were: LOC646329 Forward, CTTGGGGATCCTCTGTACGC; LOC646329 Reverse, CTTCGGTATCCTGATGTAGGTGT.

Internally controlled, relative growth assays were performed separately. Triplicates cultures of dCas9-KRAB U87 cells were transduced at 40–50 % infection rate with lentiviruses harboring sgRNAs. The proportion of cells that were BFP^hi^ ,indicating sgRNA expression, in each population was measured at every passage (every other day) by an LSR II flow cytometer (BD). These proportions were internally normalized to values at 5 days, when all infected cells reached full sgRNA expression, and then compared to cells infected with non-targeting control sgRNAs, which demonstrated stable expression of the sgRNA containing vector.

### Availability of supporting data

All sequencing data, bulk RNA-seq alignment signal, reference transcriptome GTF files, and expression tables are deposited in GSE71315. Glioblastoma cell line U87 RNA-seq data were obtained from GSE29738. Single-cell RNA-seq libraries from the 50 GW16 and GW21 samples and the 46 K562 cells were obtained from SRP041736.

## Additional files

Additional file 1: Table S1. Related to Figs. 1 and 3. Sample and sequencing statistics. Developmental stages, number of reads sequenced and aligned for each bulk RNA-seq and single-cell RNA-seq sample (separate tabs in xls file). Single-cell RNA-seq statistics also include number of unique genes and ERCC Spike-In Control species detected, and how many reads were assigned to either. (XLS 44 kb) Additional file 2: Figure S1. Computational pipeline of lncRNA identification. **A** ROC analysis of coverage thresholds required to fully annotate known transcripts in poly(A) and total RNA-seq data. Coverage thresholds were selected for which false positive rates were 0.05, corresponding to the optimal balance of false positive and true positive rates. **B** *Venn diagrams* of novel transcripts classified as protein coding by CPC, CPAT, and Pfam search. The union of the transcripts were annotated TUCPs. **C** Number of novel poly(A) lncRNAs expressed in the Full (*red*) and Stringent (*blue*) references compared to Cabili et al. 2011, Ensembl 75/GENCODE 19, Hangauer et al. [7], and Iyer et al. [6] (MiTranscriptome). **D** Maximum expression levels of transcripts described in the Full (*white*) and Stringent (*gray*) references derived total RNA-seq across all samples. TPM, Transcripts per Million. **E** Numbers of expressed mRNAs, lncRNAs, and TUCPs during neocortex development in bulk tissues, according to total RNA-seq libraries. **F** Top gene ontology terms for differentially expressed mRNAs (*left*) and mRNA neighbors of lncRNAs/TUCPs (*right*). (PDF 165 kb) Additional file 3: Table S2. Related to Fig. 1. Expression table of all bulk tissue samples and genes (including known and novel lncRNAs) according to the polyA Full transcriptome reference. Expression values are in TPM (Transcripts per Million). (XLS 9972 kb) Additional file 4: Table S3. Related to Figure S1. Table of novel lncRNAs not annotated in Ensembl release 75. Gene names and coordinates are in BED format. These lncRNAs were the set of novel lncRNAs annotated in the polyA full reference, which includes both multi-exon and single exon lncRNAs. (XLS 1429 kb) Additional file 5: Figure S2. Genomic characteristics of lncRNAs expressed in human neocortex development. **A** Cumulative distributions transcript length of mRNAs, Ensembl lncRNAs, and lncRNAs/TUCPs in the Full and Stringent references not annotated in Ensembl (novel). **B** Cumulative distributions of distances between mRNAs or lncRNAs/TUCPs to their closest mRNA neighbors. Novel lncRNAs/TUCPs were farther from mRNAs than mRNAs were to their neighboring mRNAs. **C** Histograms of exon counts for mRNA or lncRNA/TUCP transcripts. **D** Histograms of number of unique isoforms for each mRNA or lncRNA/TUCP gene. **E** Cumulative distributions of mean exonic PhastCons conservation scores for mRNAs or lncRNAs/TUCPs. All lncRNAs/TUCPs were less conserved than mRNAs, but were more conserved than repeat regions. *Left panels* represent results using polyA RNA data, *right panels* represent results using total RNA data. (PDF 211 kb) Additional file 6: Table S4. Related to Fig. 2a. Expression table of all bulk tissue samples and differentially expressed genes, using polyA data. Expression values are in TPM (Transcripts per Million). (XLS 336 kb) Additional file 7: Figure S3. Identification of lncRNAs enriched in total RNA-seq. Smooth scatter plot comparisons of mRNA (*left*) and lncRNA (*right*) expression levels between polyA RNA-seq and total RNA-seq in all eight samples. Genes not expressed either polyA or total RNA-seq were omitted. Fifty-eight mRNAs and 85 lncRNAs were consistently >10-fold enriched in total RNA-seq across all samples. Only one gene, NDUFC2-KCTD14, was identified as consistently enriched in the polyA selected RNA-seq. *Red diagonals* represent 10-fold enrichment in either total (upper) or polyA (lower) fractions. *Red triangles* represent histone subunits enriched >10-fold in total RNA. TPM, Transcripts per Million. (PDF 1053 kb) Additional file 8: Table S5. Related to Fig. 2c. Expression table of all bulk tissue samples showing genes enriched in either total RNA-seq or polyA RNA-seq. Both polyA and total RNA-seq data are represented for each tissue sample. Expression values are in TPM (Transcripts per Million). (XLS 58 kb) Additional file 9: Figure S4. Summary statistics and quality control of single-cell RNA-seq. *A* Distribution of numbers of genes detected in each of the 276 neocortex single cells analyzed. The >1000 genes detected threshold was selected due to rapid decay of complexity below 1000 genes, potentially due to failed lysis of some cells. **B** Distribution of numbers of ERCC species detected in each of the 276 neocortex single cells analyzed. The set of cells at 0 ERCCs represents single cell libraries previously sequenced and analyzed in Pollen et al. [32], which lack ERCCs. **C** Number of reads mapping to genes (*red*) and ERCC species (*blue*) in each of the 276 neocortex single cells. **D** Identification of highly variable genes (*red*; FDR <0.05; Chi-squared distribution) used for cell type inference by modeling technical noise of ERCC Spike-In Controls (*blue circles*). *Blue curve* represents a gamma generalized linear model relating the coefficients of variation squared for ERCCs as a function of mean normalized counts. *Dotted red curve* represents 50 % biological variance above technical noise. **E** Detection rate of ERCC Spike-In Control RNA at predetermined quantities of spikes across 226 neocortex single cells. *Red dotted line* represents 74 % mean detection rate at two copies per cell. **F** Linear regression model relating size factor normalized counts to ERCC molecule quantity. **G** Detection rate of ERCC Spike-In Control RNA at predetermined quantities of spikes across 46 K562 single cells. *Red dotted line* represents 62 % mean detection rate at two copies per cell. **H** Linear regression model relating normalized counts to ERCC molecule quantity in K562 cells. **I** Transcript lengths of lncRNAs (*blue*) and mRNAs (*red*). Median lncRNA length was 0.40-fold that of median mRNA length. The omission of length normalization in single-cell data therefore does not artificially overestimate lncRNA abundance. **J** Pearson correlation coefficients between ERCC molecule quantity and various expression metrics in 46 K562 single cells. CPM, Counts per Million mapped reads; FPKM, Fragments per Kilobase per Million mapped reads; TPM, Transcripts per Million. Count based metrics (Counts, CPM) outperformed length-normalized metrics (FPKM, TPM) in single-cell RNA-seq data prepared using the SMARTer method. (PDF 219 kb) Additional file 10: Table S6. Related to Fig. 3. Single cell expression table. A total of 276 single cells from human neocortex development and all genes (including known and novel lncRNAs) that pass the minimum expression threshold (Methods). Expression values are in size factor normalized counts, according to DESeq. (XLS 31786 kb) Additional file 11: Figure S5. Expression of lncRNAs and mRNAs in single cells and whole tissues. **A** Distributions of non-zero lncRNA (*blue*) and mRNA (*red*) expression in 276 single cells from neocortex. The median lncRNA expression and the median mRNA expression for each cell was compared as a ratio and summarized in Fig. 3b. **B** Distributions of non-zero lncRNA (*blue*) and mRNA (*red*) expression in eight bulk tissue RNA-seq samples using the same set of 1400 lncRNAs and 10929 mRNAs as in (A). **C** Distributions of non-zero lncRNA (*blue*) and mRNA (*red*) expression in in silico merged neocortex single cells. **D** Proportion of single neocortex cells that expressed each lncRNA (*blue*) and mRNA (*red*), binned by their expression levels in bulk tissues. **E** Distributions of housekeeping gene and lncRNA (**F**) expression levels in neocortex single cells, binned by log2(Normalized Counts + 1). Colors represent number of cells in each bin. Abundant lncRNAs were ranked by their median expression levels across 276 neocortex single cells. (PDF 257 kb) Additional file 12: Figure S6. Single cell transcriptomics of lncRNA expression in K562 cell cultures. **A** Distributions of median lncRNA expression to median mRNA expression ratios (lncRNA:mRNA) in populations, in silico merged single cells, and single cells from K562 cultures. **B** Proportion of K562 cells that expressed each lncRNA (*blue*) and mRNA (*red*), separated by maximum expression in single cells. **C** Same as in (B) but grouped by maximum expression quantile. **D** Distributions of non-zero lncRNA (*blue*) and mRNA (*red*) expression in 46 single K562 cells. *Green squares*, housekeeping genes; *black triangles*, ERCC Spike-In Controls. (PDF 454 kb) Additional file 13: Figure S7. Hierarchical clustering of neocortex single cells. Results of complete linkage hierarchical clustering of 276 neocortex single cells, using the top 500 genes ranked by the highest absolute values of gene loading scores across the first four principal components following PCA. Endo, endothelial; RG, radial glia; DRG, dividing radial glia; IPC, intermediate progenitor cell; NN, newborn neurons; MN, maturing neurons; INTER, interneurons. (PDF 51 kb) Additional file 14: Table S7. Related to Fig. 4. Table of 105 cell type-specific mRNAs (*1st tab*) and lncRNAs (*2nd tab*) and their expression enrichment scores, which are log2 transformed, for each of the seven cell type clusters. (XLS 42 kb) Additional file 15: Figure S8. Abundant expression of cell type-specific lncRNAs in single cells. **A** Distributions of expression levels of cell type-specific mRNAs and (**B**) lncRNAs in single neocortex cells, binned by log2(Normalized Counts + 1). Heatmap colors represent number of cells in each bin. Genes were ranked by their median expression levels across 276 neocortex single cells. Columns adjacent to gene names represent cell type assignments. **C** Distributions of maximum log odds ratios for cell type-specific gene expression at 75%ile threshold for each lncRNA (*blue*) and mRNA (*red*) at different quantiles of expression. **D** Cumulative distributions of cell type specificity scores for lncRNAs expressed at various levels in bulk tissues. Endo/*red*, endothelial; RG/*blue*, radial glia; DRG/*green*, dividing radial glia; IPC/*purple*, intermediate progenitor cell; NN/*orange*, newborn neurons; MN/*yellow*, maturing neurons; INTER/*brown*, interneurons. (PDF 120 kb) Additional file 16: Figure S9. Gene co-expression analysis of cell type-specific lncRNAs in single cells. **A** Matrix of Pearson correlation coefficients between cell type-specific lncRNAs and the top 10 % most correlated or anticorrelated mRNAs across single cells. **B** Gene ontology terms for gene clusters identified in the correlation matrix. Cluster nodes are labeled with *red circles*. Header bar colors for lncRNAs: *red*, endothelial; *blue*, radial glia; *green*, dividing radial glia; *purple*, intermediate progenitor cell; *orange*, newborn neurons; *yellow*, maturing neurons; *brown*, interneurons. (PDF 211 kb) Additional file 17: Figure S10. Immunohistochemistry of maturing neuron marker ADRA2A. Immunohistochemistry of maturing neuron marker protein ADRA2A (*left*) compared to in situ hybridization against maturing neuron lncRNA *LINC00599* (*right*, reproduced from Fig. 5). (PDF 952 kb)
