# Supplementary material for: Single-cell analysis of long non-coding RNAs in the developing human neocortex
Source: Genome Biol. 2016 Apr 14;17:67. doi: 10.1186/s13059-016-0932-1 (PMC4831157; doi:10.1186/s13059-016-0932-1)
Supplement: Additional file 2: Figure S1. — Computational pipeline of lncRNA identification. A ROC analysis of coverage thresholds required to fully annotate known transcripts in poly(A) and total RNA-seq data. Coverage thresholds were selected for which false positive rates were 0.05, corresponding to the optimal balance of false positive and true positive rates. B Venn diagrams of novel transcripts classified as protein coding by CPC, CPAT, and Pfam search. The union of the transcripts were annotated TUCPs. C Number of novel poly(A) lncRNAs expressed in the Full (red) and Stringent (blue) references compared to Cabili et al. 2011, Ensembl 75/GENCODE 19, Hangauer et al. [7], and Iyer et al. [6] (MiTranscriptome). D Maximum expression levels of transcripts described in the Full (white) and Stringent (gray) references derived total RNA-seq across all samples. TPM, Transcripts per Million. E Numbers of expressed mRNAs, lncRNAs, and TUCPs during neocortex development in bulk tissues, according to total RNA-seq libraries. F Top gene ontology terms for differentially expressed mRNAs (left) and mRNA neighbors of lncRNAs/TUCPs (right). (PDF 165 kb) [file 13059_2016_932_MOESM2_ESM.pdf]

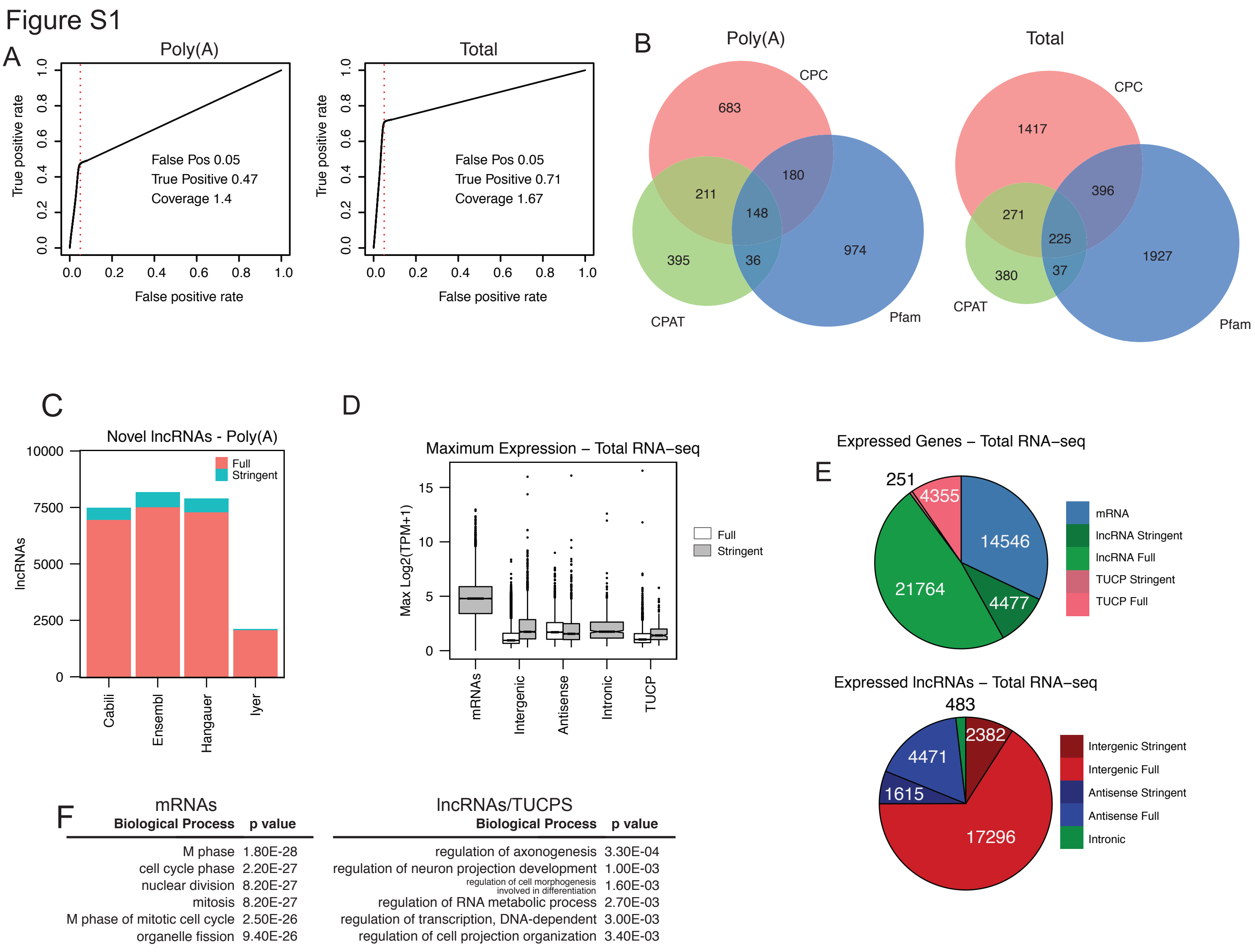

mRNAs

Biological Process	p value
M phase	1.80E-28
cell cycle phase	2.20E-27
nuclear division	8.20E-27
mitosis	8.20E-27
M phase of mitotic cell cycle	2.50E-26
organelle fission	9.40E-26

lncRNAs/TUCPS

Biological Process	p value
regulation of axonogenesis	3.30E-04
regulation of neuron projection development	1.00E-03
regulation of cell morphogenesis involved in differentiation	1.60E-03
regulation of RNA metabolic process	2.70E-03
regulation of transcription, DNA-dependent	3.00E-03
regulation of cell projection organization	3.40E-03

Figure S1. Computational pipeline of lncRNA identification

A) ROC analysis of coverage thresholds required to fully annotate known transcripts in poly(A) and total RNA-seq data. Coverage thresholds were selected for which false positive rates were 0.05, corresponding to the optimal balance of false positive and true positive rates. B) Venn diagrams of novel transcripts classified as protein coding by CPC, CPAT, and Pfam search. The union of the transcripts were annotated TUCPs. C) Number of novel poly(A) lncRNAs expressed in the Full (red) and Stringent (blue) references compared to Cabili et. al. 2013, Ensembl 75/GENCODE 19, Hangauer et. al. 2013, and Iyer et. al. 2015 (MiTranscriptome). D) Maximum expression levels of transcripts described in the Full (white) and Stringent (gray) references derived total RNA-seq across all samples. TPM, Transcripts per Million E) Numbers of expressed mRNAs, lncRNAs, and TUCPs during neocortex development in bulk tissues, according to total RNA-seq libraries. F) Top gene ontology terms for differentially expressed mRNAs (left) and mRNA neighbors of lncRNAs/TUCPs (right).
