# Supplementary material for: Single-cell analysis of long non-coding RNAs in the developing human neocortex
Source: Genome Biol. 2016 Apr 14;17:67. doi: 10.1186/s13059-016-0932-1 (PMC4831157; doi:10.1186/s13059-016-0932-1)
Supplement: Additional file 5: Figure S2. — Genomic characteristics of lncRNAs expressed in human neocortex development. A Cumulative distributions transcript length of mRNAs, Ensembl lncRNAs, and lncRNAs/TUCPs in the Full and Stringent references not annotated in Ensembl (novel). B Cumulative distributions of distances between mRNAs or lncRNAs/TUCPs to their closest mRNA neighbors. Novel lncRNAs/TUCPs were farther from mRNAs than mRNAs were to their neighboring mRNAs. C Histograms of exon counts for mRNA or lncRNA/TUCP transcripts. D Histograms of number of unique isoforms for each mRNA or lncRNA/TUCP gene. E Cumulative distributions of mean exonic PhastCons conservation scores for mRNAs or lncRNAs/TUCPs. All lncRNAs/TUCPs were less conserved than mRNAs, but were more conserved than repeat regions. Left panels represent results using polyA RNA data, right panels represent results using total RNA data. (PDF 211 kb) [file 13059_2016_932_MOESM5_ESM.pdf]

Figure S2

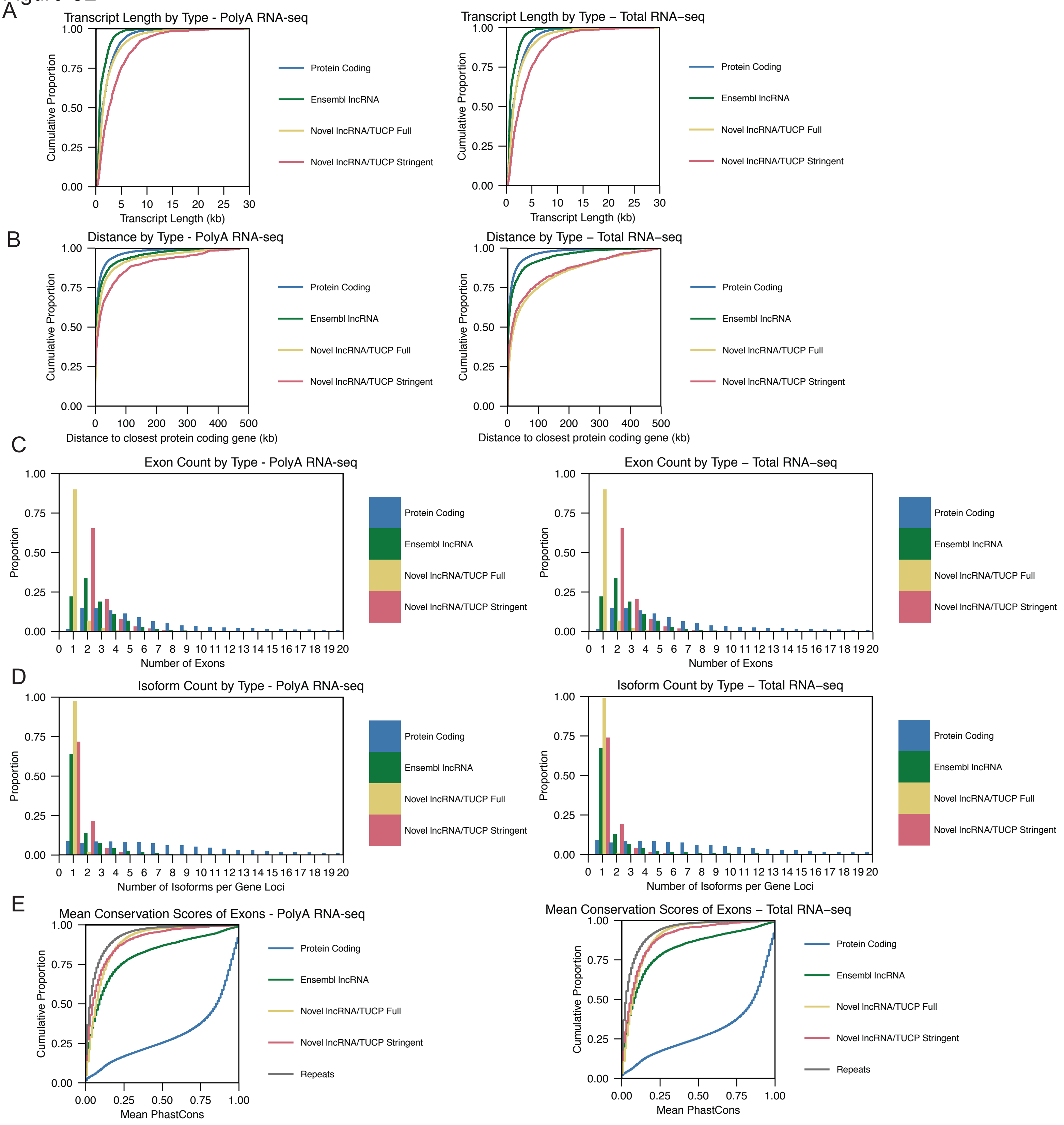

Figure S2. Genomic characteristics of lncRNAs expressed in human neocortex development
A) Cumulative distributions transcript length of mRNAs, Ensembl lncRNAs, and lncRNAs/TUCPs in the Full and Stringent references not annotated in Ensembl (novel). B) Cumulative distributions of distances between mRNAs or lncRNAs/TUCPs to their closest mRNA neighbors. Novel lncRNAs/TUCPs were farther from mRNAs than mRNAs were to their neighboring mRNAs. C) Histograms of exon counts for mRNA or lncRNA/TUCP transcripts. D) Histograms of number of unique isoforms for each mRNA or lncRNA/TUCP gene. E) Cumulative distributions of mean exonic PhastCons conservation scores for mRNAs or lncRNAs/TUCPs. All lncRNAs/TUCPs were less conserved than mRNAs, but were more conserved than repeat regions. Left panels represent results using polyA RNA data, right panels represent results using total RNA data.
