# Supplementary material for: Single-cell analysis of long non-coding RNAs in the developing human neocortex
Source: Genome Biol. 2016 Apr 14;17:67. doi: 10.1186/s13059-016-0932-1 (PMC4831157; doi:10.1186/s13059-016-0932-1)
Supplement: Additional file 7: Figure S3. — Identification of lncRNAs enriched in total RNA-seq. Smooth scatter plot comparisons of mRNA (left) and lncRNA (right) expression levels between polyA RNA-seq and total RNA-seq in all eight samples. Genes not expressed either polyA or total RNA-seq were omitted. Fifty-eight mRNAs and 85 lncRNAs were consistently >10-fold enriched in total RNA-seq across all samples. Only one gene, NDUFC2-KCTD14, was identified as consistently enriched in the polyA selected RNA-seq. Red diagonals represent 10-fold enrichment in either total (upper) or polyA (lower) fractions. Red triangles represent histone subunits enriched >10-fold in total RNA. TPM, Transcripts per Million. (PDF 1053 kb) [file 13059_2016_932_MOESM7_ESM.pdf]

Figure S3

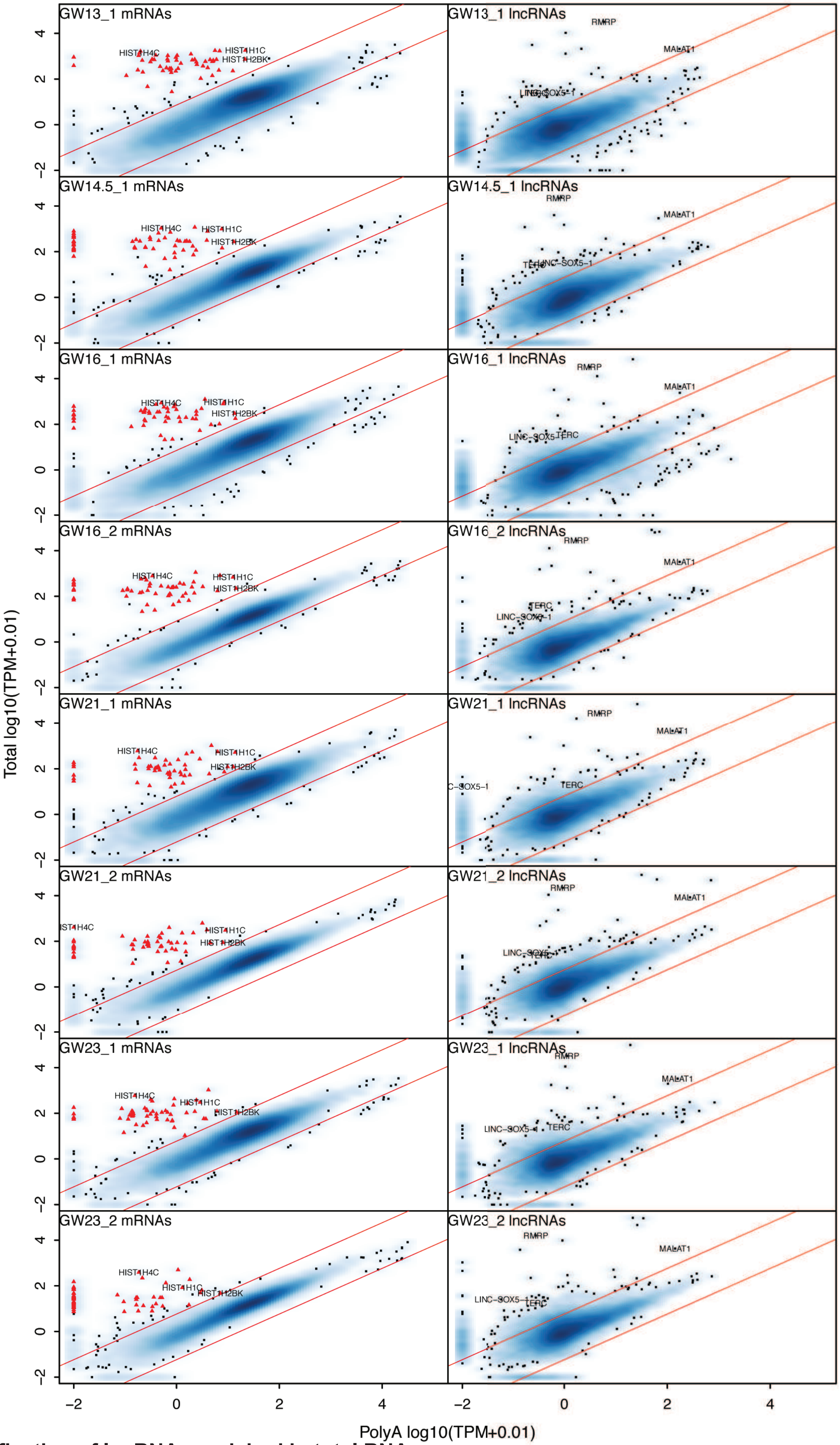

Figure S3. Identification of lncRNAs enriched in total RNA-seq

Smooth scatter plot comparisons of mRNA (left) and lncRNA (right) expression levels between polyA RNA-seq and total RNA-seq in all 8 samples. Genes not expressed either polyA or total RNA-seq were omitted. 58 mRNAs and 85 lncRNAs were consistently > 10 fold enriched in total RNA-seq across all samples. Only one gene, NDUFC2-KCTD14, was identified as consistently enriched in the polyA selected RNA-seq. Red diagonals represent 10 fold enrichment in either total (upper) or polyA (lower) fractions. Red triangles, histone subunits enriched > 10 fold in total RNA. TPM, Transcripts per Million.
