# Supplementary material for: Single-cell analysis of long non-coding RNAs in the developing human neocortex
Source: Genome Biol. 2016 Apr 14;17:67. doi: 10.1186/s13059-016-0932-1 (PMC4831157; doi:10.1186/s13059-016-0932-1)
Supplement: Additional file 9: Figure S4. — Summary statistics and quality control of single-cell RNA-seq. A Distribution of numbers of genes detected in each of the 276 neocortex single cells analyzed. The >1000 genes detected threshold was selected due to rapid decay of complexity below 1000 genes, potentially due to failed lysis of some cells. B Distribution of numbers of ERCC species detected in each of the 276 neocortex single cells analyzed. The set of cells at 0 ERCCs represents single cell libraries previously sequenced and analyzed in Pollen et al. [32], which lack ERCCs. C Number of reads mapping to genes (red) and ERCC species (blue) in each of the 276 neocortex single cells. D Identification of highly variable genes (red; FDR <0.05; Chi-squared distribution) used for cell type inference by modeling technical noise of ERCC Spike-In Controls (blue circles). Blue curve represents a gamma generalized linear model relating the coefficients of variation squared for ERCCs as a function of mean normalized counts. Dotted red curve represents 50 % biological variance above technical noise. E Detection rate of ERCC Spike-In Control RNA at predetermined quantities of spikes across 226 neocortex single cells. Red dotted line represents 74 % mean detection rate at two copies per cell. F Linear regression model relating size factor normalized counts to ERCC molecule quantity. G Detection rate of ERCC Spike-In Control RNA at predetermined quantities of spikes across 46 K562 single cells. Red dotted line represents 62 % mean detection rate at two copies per cell. H Linear regression model relating normalized counts to ERCC molecule quantity in K562 cells. I Transcript lengths of lncRNAs (blue) and mRNAs (red). Median lncRNA length was 0.40-fold that of median mRNA length. The omission of length normalization in single-cell data therefore does not artificially overestimate lncRNA abundance. J Pearson correlation coefficients between ERCC molecule quantity and various expression metrics in 46 K562 single cells. CPM, [file 13059_2016_932_MOESM9_ESM.pdf]

Figure S4

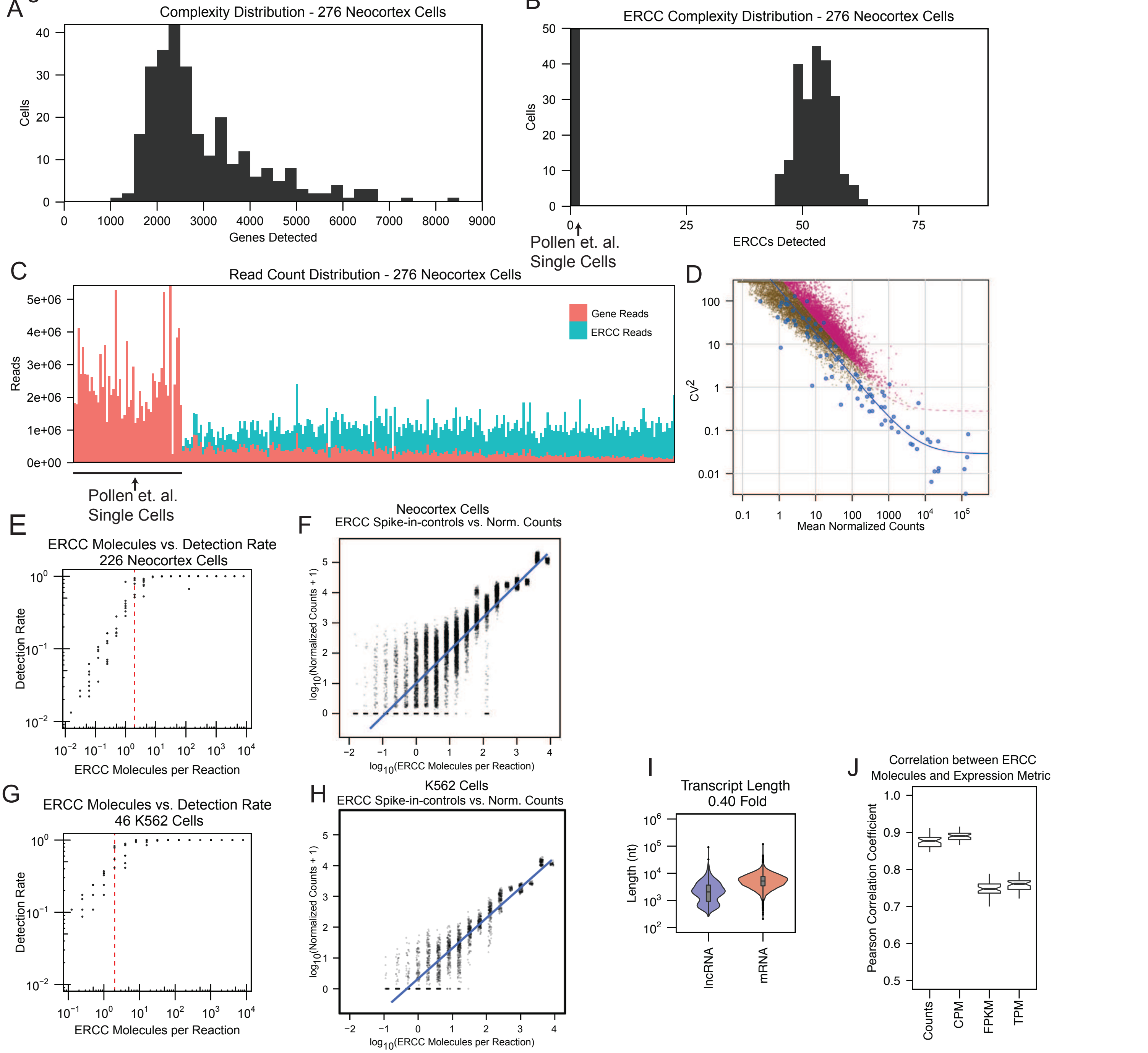

Figure S4. Summary statistics and quality control of single cell RNA-seq

A) Distribution of numbers of genes detected in each of the 276 neocortex single cells analyzed. The >1000 genes detected threshold was selected due to rapid decay of complexity below 1000 genes, potentially due to failed lysis of some cells. B) Distribution of numbers of ERCC species detected in each of the 276 neocortex single cells analyzed. The set of cells at 0 ERCCs represents single cell libraries previously sequenced and analyzed in Pollen et. al. 2014, which lack ERCCs. C) Number of reads mapping to genes (red) and ERCC species (blue) in each of the 276 neocortex single cells. D) Identification of highly variable genes (red; FDR < 0.05; Chi-squared distribution) used for cell type inference by modeling technical noise of ERCC Spike-In Controls (blue circles). Blue curve represents a gamma generalized linear model relating the coefficients of variation squared for ERCCs as a function of mean normalized counts. Dotted red curve represents 50% biological variance above technical noise. E) Detection rate of ERCC Spike-In Control RNA at predetermined quantities of spikes across 226 neocortex single cells. Red dotted line represents 74% mean detection rate at 2 copies per cell. F) Linear regression model relating size factor normalized counts to ERCC molecule quantity. G) Detection rate of ERCC Spike-In Control RNA at predetermined quantities of spikes across 46 K562 single cells. Red dotted line represents 62% mean detection rate at 2 copies per cell. H) Linear regression model relating normalized counts to ERCC molecule quantity in K562 cells. I) Transcript lengths of lncRNAs (blue) and mRNAs (red). Median lncRNA length was 0.40 fold that of median mRNA length. The omission of length normalization in single cell data therefore does not artificially overestimate lncRNA abundance. J) Pearson correlation coefficients between ERCC molecule quantity and various expression metrics in 46 K562 single cells. CPM, Counts per Million mapped reads. FPKM, Fragments per Kilobase per Million mapped reads. TPM, Transcripts per Million. Count based metrics (Counts, CPM) outperformed length-normalized metrics (FPKM, TPM) in single cell RNA-seq data prepared using the SMARTer method.
