# Supplementary material for: Single-cell analysis of long non-coding RNAs in the developing human neocortex
Source: Genome Biol. 2016 Apr 14;17:67. doi: 10.1186/s13059-016-0932-1 (PMC4831157; doi:10.1186/s13059-016-0932-1)
Supplement: Additional file 11: Figure S5. — Expression of lncRNAs and mRNAs in single cells and whole tissues. A Distributions of non-zero lncRNA (blue) and mRNA (red) expression in 276 single cells from neocortex. The median lncRNA expression and the median mRNA expression for each cell was compared as a ratio and summarized in Fig. 3b. B Distributions of non-zero lncRNA (blue) and mRNA (red) expression in eight bulk tissue RNA-seq samples using the same set of 1400 lncRNAs and 10929 mRNAs as in (A). C Distributions of non-zero lncRNA (blue) and mRNA (red) expression in in silico merged neocortex single cells. D Proportion of single neocortex cells that expressed each lncRNA (blue) and mRNA (red), binned by their expression levels in bulk tissues. E Distributions of housekeeping gene and lncRNA (F) expression levels in neocortex single cells, binned by log2(Normalized Counts + 1). Colors represent number of cells in each bin. Abundant lncRNAs were ranked by their median expression levels across 276 neocortex single cells. (PDF 257 kb) [file 13059_2016_932_MOESM11_ESM.pdf]

Figure S5

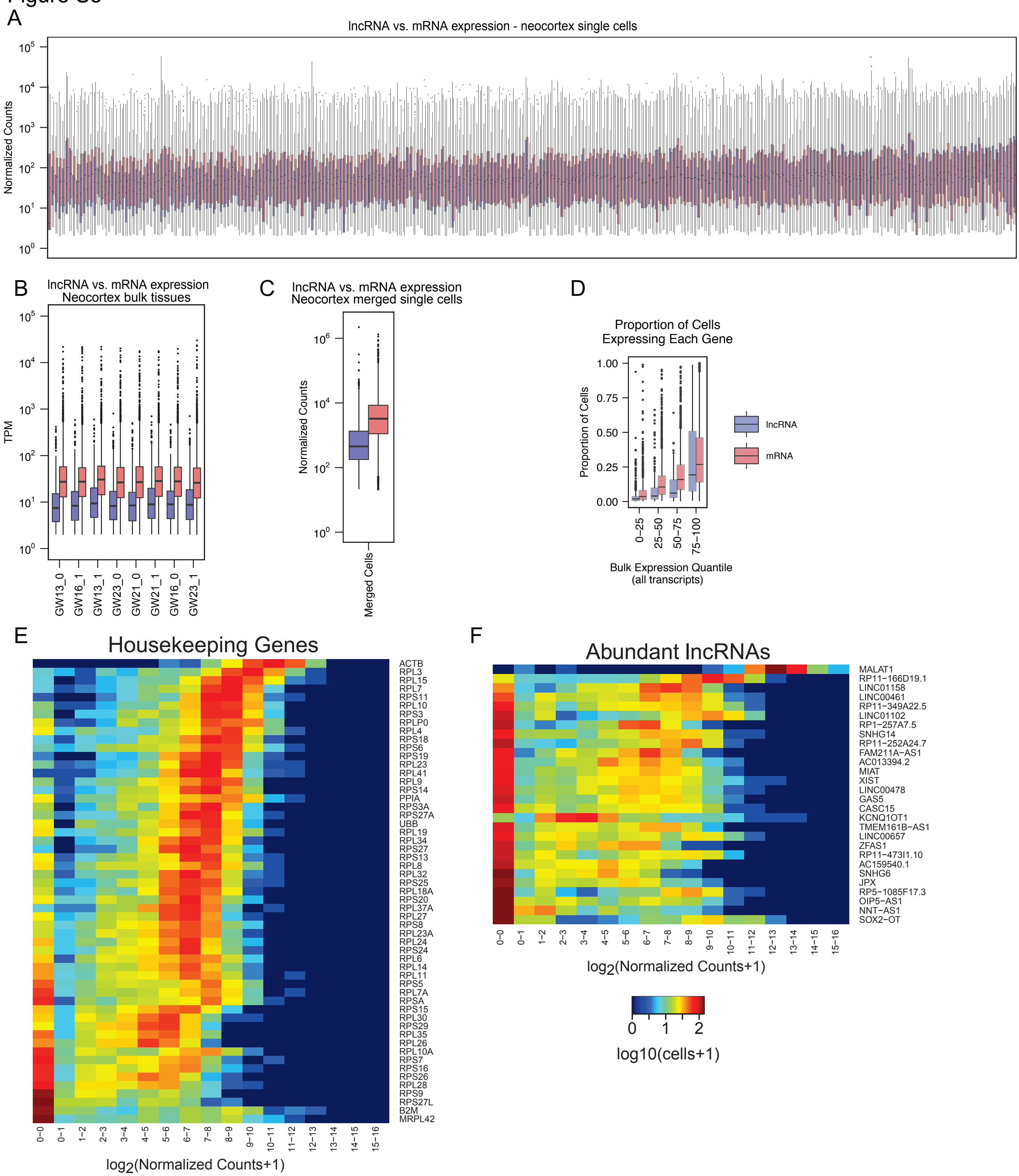

Figure S5. Expression of lncRNAs and mRNAs in single cells and whole tissues

A) Distributions of non-zero lncRNA (blue) and mRNA (red) expression in 276 single cells from neocortex. The median lncRNA expression and the median mRNA expression for each cell was compared as a ratio and summarized in Figure 3B. B) Distributions of non-zero lncRNA (blue) and mRNA (red) expression in 8 bulk tissue RNA-seq samples using the same set of 1400 lncRNAs and 10929 mRNAs as in A). C) Distributions of non-zero lncRNA (blue) and mRNA (red) expression in in silico merged neocortex single cells. D) Proportion of single neocortex cells that expressed each lncRNA (blue) and mRNA (red), binned by their expression levels in bulk tissues. E) Distributions of housekeeping gene and lncRNA (F) expression levels in neocortex single cells, binned by log2(Normalized Counts + 1). Colors represent number of cells in each bin. Abundant lncRNAs were ranked by their median expression levels across 276 neocortex single cells.
