# Supplementary material for: Single-cell analysis of long non-coding RNAs in the developing human neocortex
Source: Genome Biol. 2016 Apr 14;17:67. doi: 10.1186/s13059-016-0932-1 (PMC4831157; doi:10.1186/s13059-016-0932-1)
Supplement: Additional file 12: Figure S6. — Single cell transcriptomics of lncRNA expression in K562 cell cultures. A Distributions of median lncRNA expression to median mRNA expression ratios (lncRNA:mRNA) in populations, in silico merged single cells, and single cells from K562 cultures. B Proportion of K562 cells that expressed each lncRNA (blue) and mRNA (red), separated by maximum expression in single cells. C Same as in (B) but grouped by maximum expression quantile. D Distributions of non-zero lncRNA (blue) and mRNA (red) expression in 46 single K562 cells. Green squares, housekeeping genes; black triangles, ERCC Spike-In Controls. (PDF 454 kb) [file 13059_2016_932_MOESM12_ESM.pdf]

Figure S6

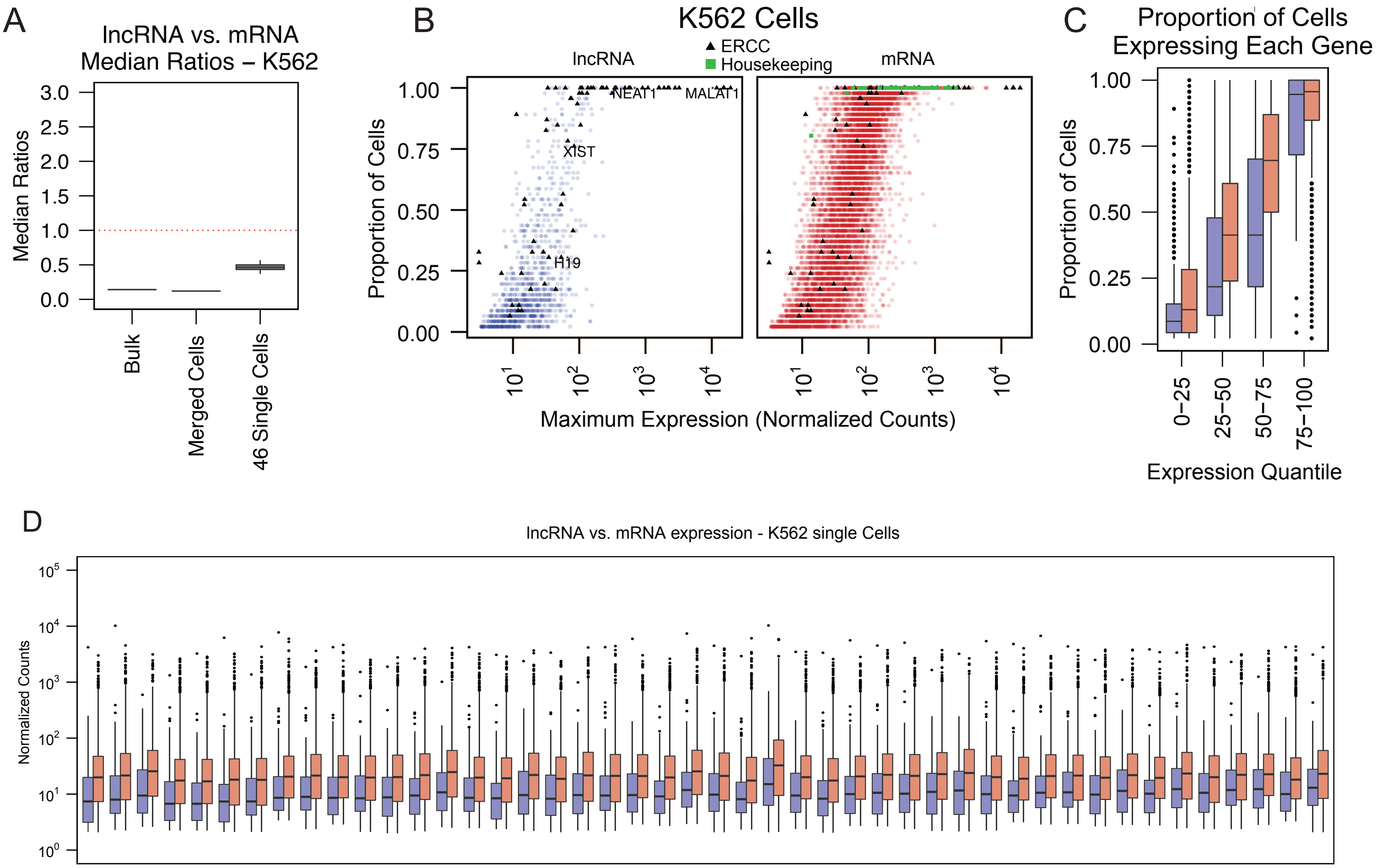

Figure S6. Single cell transcriptomics of lncRNA expression in K562 cell cultures
A) Distributions of median lncRNA expression to median mRNA expression ratios (lncRNA:mRNA) in populations, in silico merged single cells, and single cells from K562 cultures. B) Proportion of K562 cells that expressed each lncRNA (blue) and mRNA (red), separated by maximum expression in single cells. C) Same as in B) but grouped by maximum expression quantile. D) Distributions of non-zero lncRNA (blue) and mRNA (red) expression in 46 single K562 cells. Green squares, housekeeping genes. Black triangles, ERCC Spike-In Controls.
