# Supplementary material for: Single-cell analysis of long non-coding RNAs in the developing human neocortex
Source: Genome Biol. 2016 Apr 14;17:67. doi: 10.1186/s13059-016-0932-1 (PMC4831157; doi:10.1186/s13059-016-0932-1)
Supplement: Additional file 13: Figure S7. — Hierarchical clustering of neocortex single cells. Results of complete linkage hierarchical clustering of 276 neocortex single cells, using the top 500 genes ranked by the highest absolute values of gene loading scores across the first four principal components following PCA. Endo, endothelial; RG, radial glia; DRG, dividing radial glia; IPC, intermediate progenitor cell; NN, newborn neurons; MN, maturing neurons; INTER, interneurons. (PDF 51 kb) [file 13059_2016_932_MOESM13_ESM.pdf]

Figure S7

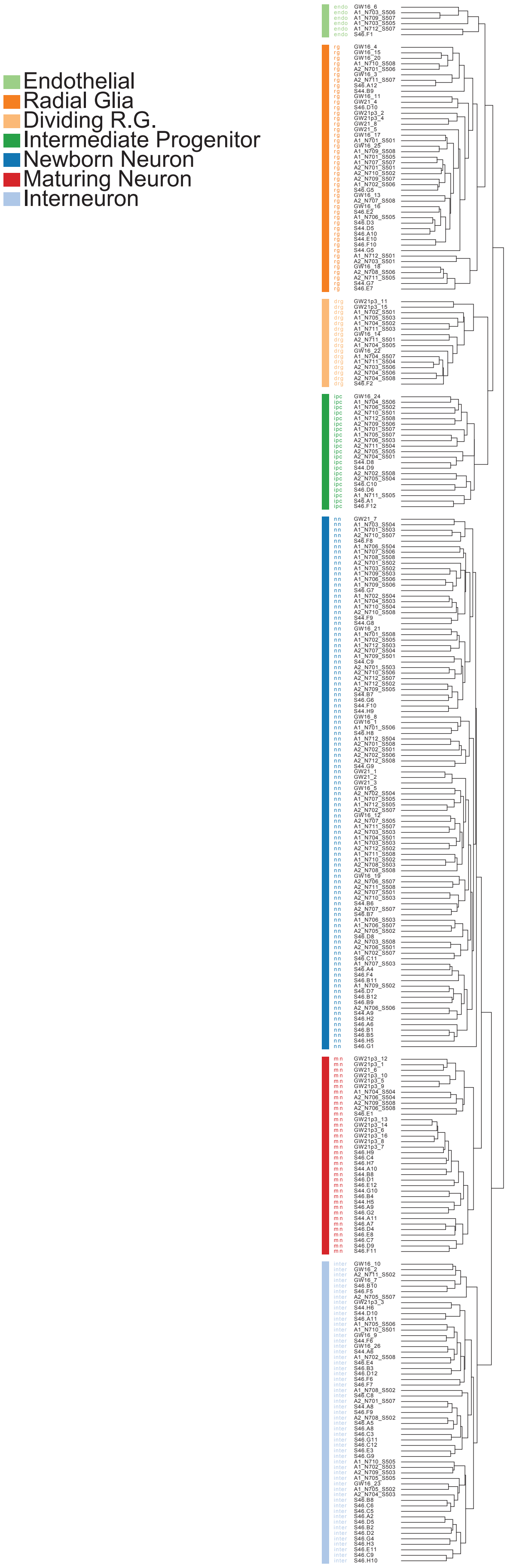

Figure S7. Hierarchical Clustering of Neocortex Single Cells

Results of complete linkage hierarchical clustering of 276 neocortex single cells, using the top 500 genes ranked by the highest absolute values of gene loading scores across the first four principal components following PCA. Endo, endothelial. RG, radial glia. DRG, dividing radial glia. IPC, intermediate progenitor cell. NN, newborn neurons. MN, maturing neurons. INTER, interneurons.
