# Supplementary material for: Single-cell analysis of long non-coding RNAs in the developing human neocortex
Source: Genome Biol. 2016 Apr 14;17:67. doi: 10.1186/s13059-016-0932-1 (PMC4831157; doi:10.1186/s13059-016-0932-1)
Supplement: Additional file 15: Figure S8. — Abundant expression of cell type-specific lncRNAs in single cells. A Distributions of expression levels of cell type-specific mRNAs and (B) lncRNAs in single neocortex cells, binned by log2(Normalized Counts + 1). Heatmap colors represent number of cells in each bin. Genes were ranked by their median expression levels across 276 neocortex single cells. Columns adjacent to gene names represent cell type assignments. C Distributions of maximum log odds ratios for cell type-specific gene expression at 75%ile threshold for each lncRNA (blue) and mRNA (red) at different quantiles of expression. D Cumulative distributions of cell type specificity scores for lncRNAs expressed at various levels in bulk tissues. Endo/red, endothelial; RG/blue, radial glia; DRG/green, dividing radial glia; IPC/purple, intermediate progenitor cell; NN/orange, newborn neurons; MN/yellow, maturing neurons; INTER/brown, interneurons. (PDF 120 kb) [file 13059_2016_932_MOESM15_ESM.pdf]

Figure S8

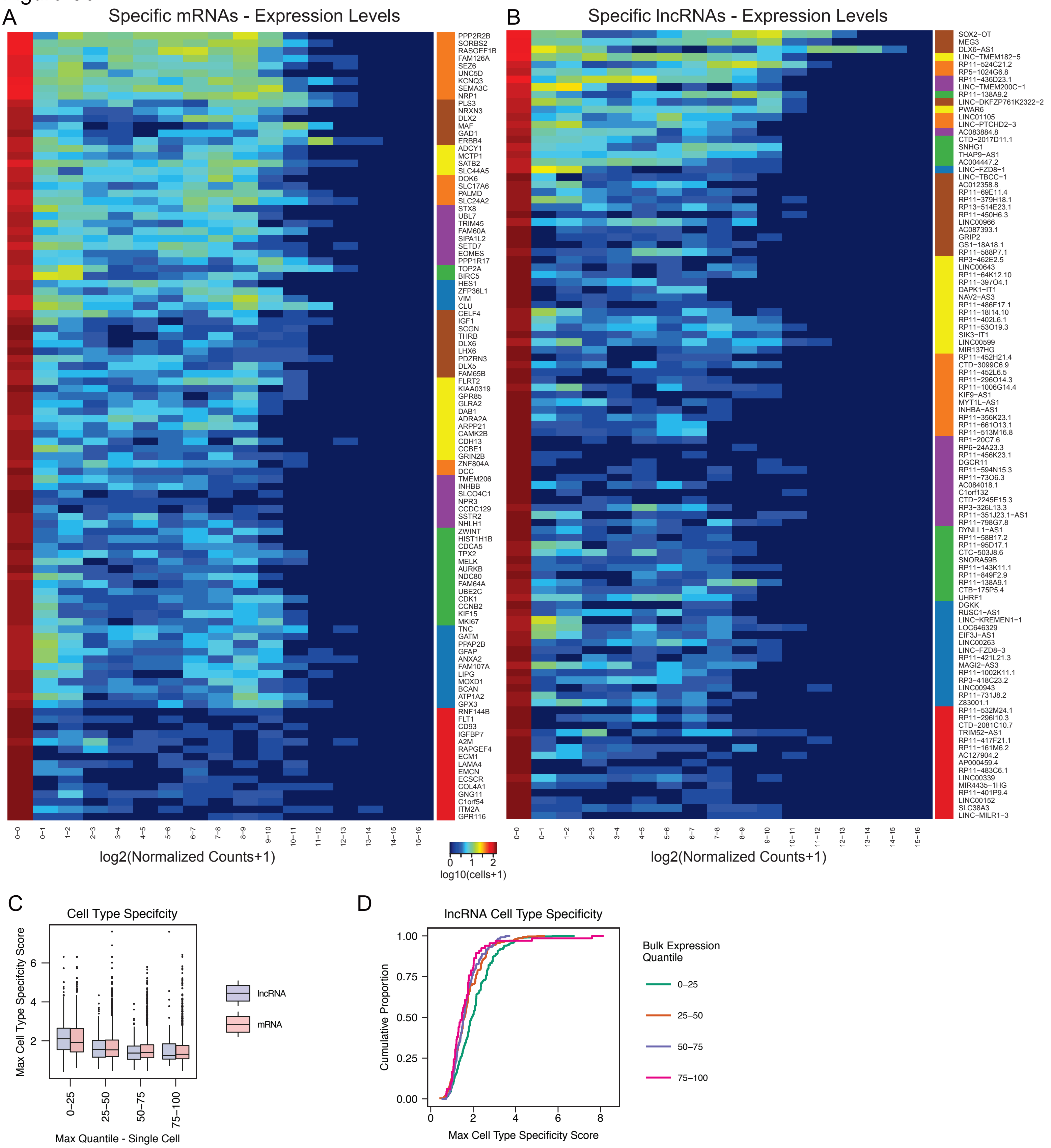

Figure S8. Abundant expression of cell type-specific lncRNAs in single cells
A) Distributions of expression levels of cell type-specific mRNAs and (B) lncRNAs in single neocortex cells, binned by log2(Normalized Counts + 1). Heatmap colors represent number of cells in each bin. Genes were ranked by their median expression levels across 276 neocortex single cells. Columns adjacent to gene names represent cell type assignments. C) Distributions of maximum log odds ratios for cell type-specific gene expression at 75%ile threshold for each lncRNA (blue) and mRNA (red) at different quantiles of expression. D) Cumulative distributions of cell type specificity scores for lncRNAs expressed at various levels in bulk tissues. Endo/red, endothelial. RG/blue, radial glia. DRG/green, dividing radial glia. IPC/purple, intermediate progenitor cell. NN/orange, newborn neurons. MN/yellow, maturing neurons. INTER/brown, interneurons.
