# Supplementary material for: Single-cell analysis of long non-coding RNAs in the developing human neocortex
Source: Genome Biol. 2016 Apr 14;17:67. doi: 10.1186/s13059-016-0932-1 (PMC4831157; doi:10.1186/s13059-016-0932-1)
Supplement: Additional file 16: Figure S9. — Gene co-expression analysis of cell type-specific lncRNAs in single cells. A Matrix of Pearson correlation coefficients between cell type-specific lncRNAs and the top 10 % most correlated or anticorrelated mRNAs across single cells. B Gene ontology terms for gene clusters identified in the correlation matrix. Cluster nodes are labeled with red circles. Header bar colors for lncRNAs: red, endothelial; blue, radial glia; green, dividing radial glia; purple, intermediate progenitor cell; orange, newborn neurons; yellow, maturing neurons; brown, interneurons. (PDF 211 kb) [file 13059_2016_932_MOESM16_ESM.pdf]

Figure S9

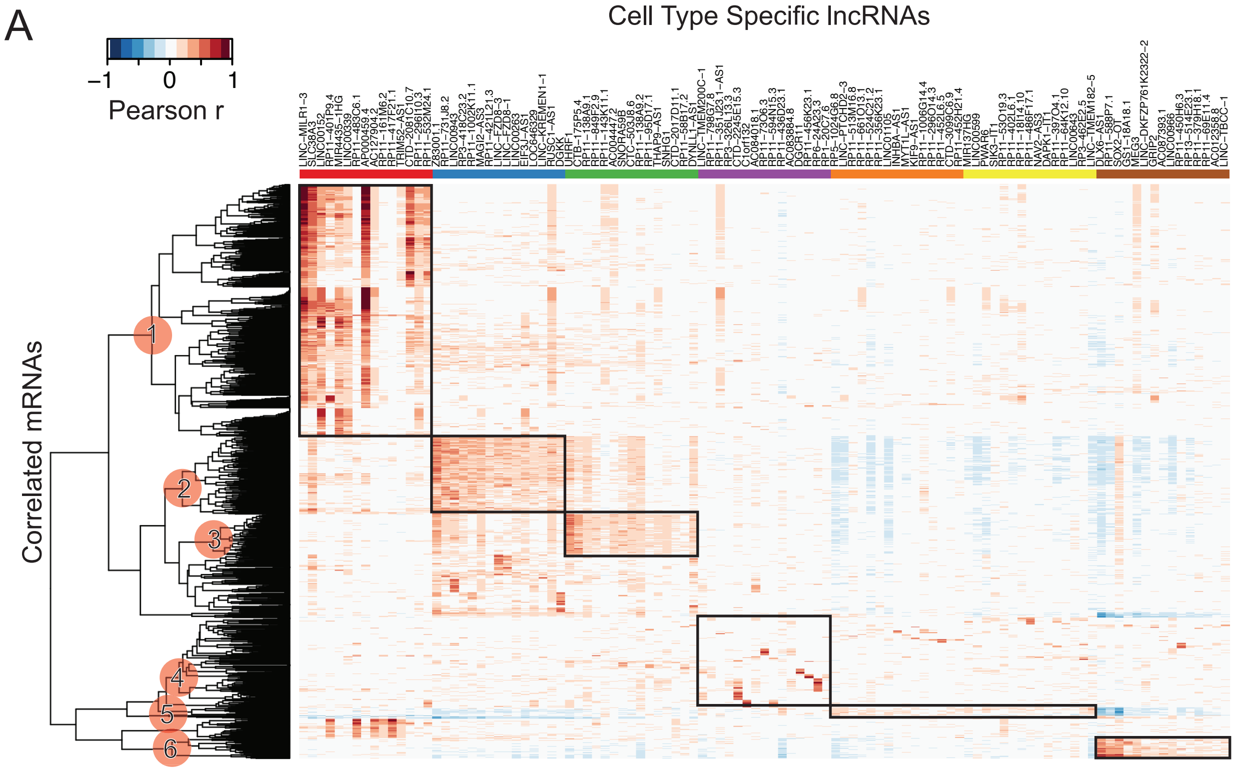

B

1

Biological Process	p value
angiogenesis	7.39E-20
regulation of vasculature development	1.68E-11
hemostasis	7.47E-11
response to oxygen levels	3.01E-10
blood coagulation	1.72E-10
coagulation	1.72E-10
regulation of angiogenesis	5.93E-11
response to decreased oxygen levels	1.58E-09
response to hypoxia	4.60E-09
extracellular matrix organization	2.45E-09

2

Biological Process	p value
negative regulation of nervous system development	8.26E-12
negative regulation of neuron differentiation	4.38E-12
negative regulation of cell development	5.66E-11
negative regulation of neurogenesis	1.77E-10
glial cell differentiation	1.52E-09
response to mechanical stimulus	4.55E-09
regulation of neuron differentiation	1.46E-07
extracellular matrix organization	7.32E-07
extracellular structure organization	7.58E-07
positive regulation of neuroblast proliferation	2.96E-06

3

Biological Process	p value
mitotic cell cycle	8.94E-35
nuclear division	5.00E-30
organelle fission	5.40E-29
mitotic nuclear division	1.40E-29
chromosome segregation	7.81E-23
regulation of cell cycle process	6.82E-13
cell cycle checkpoint	1.89E-13
chromosome organization	3.60E-13
DNA repair	3.77E-12
microtubule-based process	1.31E-10

4

Biological Process	p value
dicarboxylic acid biosynthetic process	4.83E-03
glutamine family amino acid biosynthetic process	1.25E-02
GPI anchor metabolic process	1.64E-03
regulation of triglyceride biosynthetic process	9.22E-03
glutamate metabolic process	1.88E-02
neuroblast proliferation	9.22E-03
neuromuscular synaptic transmission	1.36E-02
GPI anchor biosynthetic process	1.74E-02
positive regulation of triglyceride metabolic process	1.03E-02
positive regulation of triglyceride biosynthetic process	4.83E-03

5

Biological Process	p value
limb bud formation	4.77E-07
cardiac ventricle morphogenesis	4.82E-06
cardiac chamber morphogenesis	6.68E-06
axon extension	8.96E-06
regulation of neuron differentiation	7.58E-06
neuron projection extension	1.38E-05
positive regulation of neuron differentiation	6.30E-06
positive regulation of neurogenesis	2.22E-05
glial cell development	2.01E-05
regulation of neuron projection development	1.94E-05

6

Reactome	p value
GABA synthesis, release, reuptake and degradation	3.25E-04
Transmission across Chemical Synapses	1.97E-03
Neurotransmitter Release Cycle	1.44E-03
Nuclear Receptor transcription pathway	2.09E-03
Signaling by ERBB2	1.90E-02
Signaling by FGFR	1.87E-02
Signaling by FGFR in disease	2.21E-02
Neuronal System	5.02E-03
Downstream signal transduction	1.85E-02
Downstream signaling of activated FGFR	1.60E-02

Figure S9. Gene co-expression analysis of cell type-specific lncRNAs in single cells
A) Matrix of Pearson correlation coefficients between cell type-specific lncRNAs and the top 10% most correlated or anticorrelated mRNAs across single cells. B) Gene ontology terms for gene clusters identified in the correlation matrix. Cluster nodes are labeled with red circles. Header bar colors for lncRNAs: red, endothelial. blue, radial glia. green, dividing radial glia. purple, intermediate progenitor cell. orange, newborn neurons. yellow, maturing neurons. brown, interneurons.
