# Supplementary material for: Single-cell analysis of long non-coding RNAs in the developing human neocortex
Source: Genome Biol. 2016 Apr 14;17:67. doi: 10.1186/s13059-016-0932-1 (PMC4831157; doi:10.1186/s13059-016-0932-1)
Supplement: Additional file 17: Figure S10. — Immunohistochemistry of maturing neuron marker ADRA2A. Immunohistochemistry of maturing neuron marker protein ADRA2A (left) compared to in situ hybridization against maturing neuron lncRNA LINC00599 (right, reproduced from Fig. 5). (PDF 952 kb) [file 13059_2016_932_MOESM17_ESM.pdf]

Figure S10

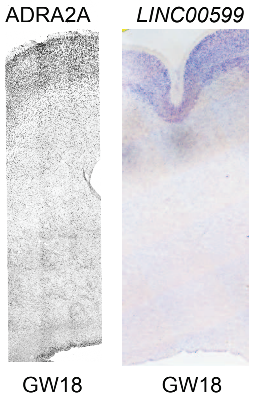

Figure S10. Immunohistochemistry of maturing neuron marker ADRA2A
Immunohistochemistry of maturing neuron marker protein ADRA2A (left) compared to in situ hybridization against maturing neuron lncRNA LINC00599 (right, reproduced from Figure 5).
